# Exploring lead-free A_2_AgRhF_6_ fluoride double perovskites for photovoltaic applications: a first-principles and device simulation study

**DOI:** 10.1039/d6ra01913g

**Published:** 2026-05-07

**Authors:** Imtiaz Ahamed Apon, Rifat Rafiu, Md. Sakib Hasan, Md. Azizur Rahman, Syeda Sayema Sumaia, Amnah Mohammed Alsuhaibani, Moamen S. Refat, Mohamed Benghanem, S. AlFaify, Noureddine Elboughdiri

**Affiliations:** a Electronics and Information Technology, University of South Wales Treforest, Pontypridd CF37 1DL UK; b Department of Material Science and Engineering, Khulna University of Engineering & Technology (KUET) Khulna-9203 Bangladesh; c Department of Electrical and Electronic Engineering, Begum Rokeya University Rangpur 5400 Bangladesh azizurrahmanatik49@gmail.com; d Department of Sports Health, College of Sport Sciences & Physical Activity, Princess Nourah bint Abdulrahman University P.O. Box 84428 Riyadh 11671 Saudi Arabia; e Department of Chemistry, College of Science, Taif University P.O. Box 11099 Taif 21944 Saudi Arabia; f Physics Department, Faculty of Science, Islamic University of Madinah Madinah 42351 Saudi Arabia mbenghanem@iu.edu.sa; g Department of Physics, College of Sciences, King Khalid University P.O. Box 960, AlQura'a Abha 61421 Saudi Arabia; h Chemical Engineering Department, College of Engineering, University of Ha'il P.O. Box 2440 81441 Ha'il Saudi Arabia

## Abstract

Lead-free fluoride double perovskites have emerged as promising candidates for stable and environmentally benign optoelectronic applications. In this work, a comprehensive first-principles and device-level investigation of A_2_AgRhF_6_ (A = K, Rb, Cs) double perovskites is performed using density functional theory (DFT) within the CASTEP framework, combined with SCAPS-1D solar cell simulations. Structural optimization confirms cubic phase stability with a systematic lattice expansion from K to Cs, while negative formation energies verify thermodynamic stability. Electronic structure analysis reveals direct band gap semiconducting behavior, with band gaps decreasing along the series due to enhanced lattice expansion and modified d–p hybridization. Effective mass calculations indicate improved electron transport with increasing A-site ionic radius, whereas hole transport remains nearly composition-independent. Optical investigations demonstrate strong ultraviolet absorption, moderate visible-light activity, high dielectric response, and pronounced plasmonic features, highlighting their suitability for UV optoelectronic and photonic applications. Charge density and population analyses reveal dominant ionic character of A-site cations and strong covalent Rh–F/Ag–F interactions, confirming that the electronic structure is primarily governed by the AgRhF_6_ octahedral framework. Mechanical stability is verified through elastic constants satisfying Born criteria, while anisotropy analysis demonstrates moderate directional dependence of elastic behavior. *Ab initio* molecular dynamics (AIMD) simulations further confirm thermal stability at elevated temperatures, validating structural robustness under operating conditions. Device simulations under AM1.5G illumination demonstrate promising photovoltaic performance with optimized absorber thickness and doping concentration, where Cs_2_AgRhF_6_ exhibits the highest power conversion efficiency among experimentally feasible compositions. This combined DFT-device-level study establishes a direct correlation between intrinsic bonding, mechanical stability, thermal robustness, and photovoltaic performance, positioning A_2_AgRhF_6_ double perovskites as promising candidates for stable lead-free solar energy conversion and advanced optoelectronic devices.

## Abstract 2

Lead-free fluoride double perovskites have emerged as promising candidates for stable and environmentally benign optoelectronic applications.

## Graphical abstract

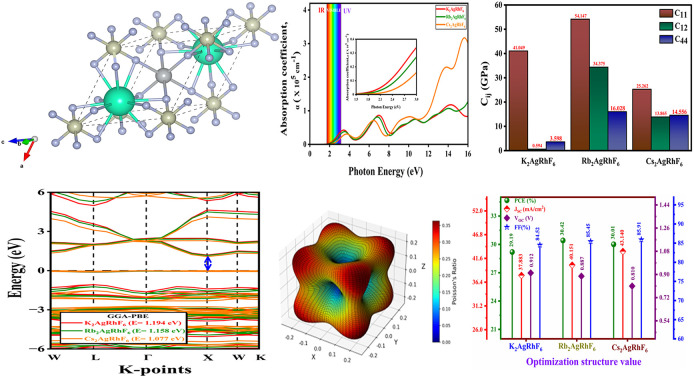

## 1 Introduction

The world is seeing a great energy crisis, resulting from increased consumption and environmental damage. It has put forth an urgent requirement for clean, efficient, and sustainable energy solutions. Perovskite solar cells (PSCs) have come forth as a great advance in photovoltaic technology because of their high-power conversion rates and ability to be used in low-cost solution-based manufacturing.^1–3^ At the base of these devices are perovskite-structured materials. Their flexible (ABX_3_) and related frameworks enable precise tuning of electronic structure, absorption, and stability *via* changes in composition.^4–6^ Also of note are lead-free double perovskites of great interest. They present high absorption rates, tunable band gaps, and better inherent stability.^6,7^ Therefore, it reduces environmental impact, unlike traditional lead-based perovskites, which may achieve over 25% efficiency but also present serious issues of lead leaching and long-term degradation.^8,9^ Double perovskites have put forth a greater range in which to optimize optoelectronic performance, and also address safety and durability issues. Moreover, they have strong structures that support tailoring of band alignment, defect tolerance, and charge transport, which is required for next-generation photovoltaic design.^10,11^ Thus, lead-free double perovskites are increasingly seen as very good options for sustainable high-efficiency solar energy conversion, and it ties material development to global environmental and energy goals. Double halide perovskites have become the top choice of lead-free materials because of their flexible makeup, environmental friendliness, and great performance in electronic and energy-related technologies. Also of note is their general formula A_2_BX_6_ or A_2_BB'X_6,_ which allows for wide substitution of cations and anions. This flexibility enables systematic changes in structural stability, band gap, charge transport, and defect chemistry for specific device applications. In particular, fluoride-based double perovskites benefit from strong ionic bonding and high chemical robustness. It makes them very attractive for use in which long-term stability and resistance to moisture or thermal stress are a must.

Recent reports on halide double perovskites have brought to light their value as non-toxic, stable, and at the same time very efficient materials for use in optoelectronic, photovoltaic and thermoelectric applications. For example, DFT studies of A_2_AgRhCl_6_ (A = Li, Na, K, Rb, Cs) reported very stable cubic structures, direct band gap semiconductor character, and large-scale light absorption, which in turn puts forward their put forth their value in photoelectric applications.^12^ Also, A_2_AgIrCl_6_ (A – Cs, Rb, K) reported cubic stability, low effective electron masses, and large-scale absorption of visible light. They validate their value as high carrier mobility materials for solar cells.^13^ Studies on Rb_2_AgMCl_6_ (M – As, Co, Rh) reported that these double perovskites have visible range band gaps (1.84–2.18 eV) and very good optical properties. For example, they include high absorption and low reflectivity, thus making them good for LEDs, photo detectors, and thermoelectric devices.^14^ Report on Cs_2_AgMX_6_ (M – In, Sb) nanocrystals, which showed a single crystalline cubic morphology and tuned chemical stability. This study gave us quantitative data on their environmental stability.^15^ Lead-free systems such as Cs_2_BiAgCl_6_ and Cs_2_BiAgBr_6_ thus reported to have indirect band gaps with very good optoelectronic properties, which reinforce the viability of bismuth-based perovskites for solar energy conversion.^16^ Also, fluoride-based A_2_AgRhF_6_ (A – Na, Rb) were very stable structurally with moderate band gaps and very good thermoelectric performance.^17^ Bismuth and antimony-based halide double perovskites (A_2_AgBX_6_, B = Bi, Sb) have been extensively reviewed as stable, lead-free photovoltaic materials with tunable optoelectronic properties.^18,19^ Mixed halide systems, such as Cs_2_AgSbCl_6−*δ*_Br_*δ*_, report tunable indirect band gaps and modified structural symmetries. It plays a great role in the control of electronic and optical behavior.^20^ Also, first-principles (*ab initio*) investigations of the LiRuPO_4_ compound reveal that structural modifications and thermal stability significantly influence its electronic properties, highlighting its potential for energy storage applications.^21^ Detailed compute-based analysis of Cs_2_AgBX_6_ (B − Bi, Sb; X – Br, Cl), suggested a strong correlation between orbital contributions, bonding characters, and optical response, reinforcing their value as non-toxic alternatives to Pb-based perovskites in opto-electronic devices.^22^ Consistent with these findings, Preeti Bhumla *et al.* reported strong lattice anharmonicity and favorable thermoelectric performance in Cs_2_BI_6_ (B = Pt, Pd, Te, Sn), attributed to the absence of polyhedral connectivity, which enhances electron–phonon coupling.^23^ Likewise, Sanchi Monga *et al.* demonstrated that anti-perovskite nitrides X_3_N_A_ (X = Mg, Ca, Sr, Ba; A = Sb, As) possess low exciton binding energies, weak electron–phonon interaction, and high carrier mobility, making them suitable for photovoltaic absorbers.^24^ As a whole, these studies put forth a very robust framework for the investigation of A_2_AgRhF_6_ double perovskites using CASTEP and SCAPS-1D in terms of their structure, electronic, and optical properties relevant to photovoltaic and optoelectronic applications.

Despite the fact that there is an increasing amount of research done on halide double perovskites, most of them have primarily focused on chloride and bromide-based systems, fluoride-based perovskites like A_2_AgRhF_6_ still are, for the most part unlooked at, unexplored in terms of their structural, electronic, mechanical, optical, and thermoelectric properties. Also, it has been the case that past studies look at only static material properties or individual aspects like band structure or optical response, which in turn leaves a gap in the full-scale evaluation of device-relevant performance parameters. To fill in this gap, the present study uses first principles density functional theory within the CASTEP framework backed up with SCAPS-1D simulations to do a system-wide analysis of A_2_AgRhF_6_ double perovskite. This we did to look at how the A site cation variation plays a role in lattice stability, electronic band structure, optical absorption, elastic properties, phonon behavior and charge distribution also at the same time we looked at how these material features play into what we see in solar cells in terms of absorber layer thickness, interface defect densities, current voltage response, quantum efficiency and overall device efficiency. By looking at both the base material science issues and device level, we present a fuller picture of fluoride-based double perovskites, which will be a path towards environmentally friendly, lead-free, high-performance optoelectronic and energy harvesting materials.

## 2 Computational method

### 2.1 First-principles calculations using CASTEP

The present study investigates the structural, electronic, optical, elastic, and mechanical properties of the double perovskite system A_2_AgRhF_6_ using first-principles density functional theory (DFT) calculations, as implemented in the Cambridge Serial Total Energy Package (CASTEP).^25^ The exchange–correlation potential was described within the framework of the generalized gradient approximation (GGA) using the Perdew–Burke–Ernzerhof (PBE) functional to capture electron exchange and correlation effects with good accuracy.^26^ A plane-wave pseudopotential method was employed, with Koelling–Harmon relativistic corrections applied to account for electron–ion interactions more precisely.^27^ Prior to the final calculations, systematic convergence tests were carried out with respect to plane-wave cutoff energy, *k*-point sampling, and number of bands to ensure the reliability and accuracy of the results. The total energy convergence criterion was set within 1 meV per atom. Based on these tests, a cutoff energy of 520 eV and a 6 × 6 × 6 Monkhorst–Pack *k*-point grid were found to yield well-converged results, with negligible variation in total energy and electronic properties upon further increase of these parameters. The number of bands was also carefully selected to ensure accurate representation of both valence and conduction states. The geometry optimizations were carried out with stringent convergence thresholds, namely an energy tolerance of 1.0 × 10^−5^ eV per atom, a maximum ionic force of 0.03 eV Å^−1^, a stress limit of 0.05 GPa, and a maximum ionic displacement of 0.001 Å, to ensure reliable equilibrium structures. The self-consistent field (SCF) calculations were performed in ultrafine mode for enhanced electronic accuracy.^28^ The Broyden–Fletcher–Goldfarb–Shanno minimization technique^29^ was used to optimize the crystalline structures by reducing Hellmann–Feynman forces until equilibrium was reached.^30^ A Monkhorst–Pack grid of 6 × 6 × 6 *k*-points was employed for Brillouin zone sampling to ensure convergence of electronic and optical properties.^31^ The valence electron configurations included in the simulations were K (3p^6^4s^1^), Rb (4p^6^5s^1^), Cs (5p^6^6s^1^), Ag (4d^10^5s^1^), Rh (4d^8^5s^1^), and F (2s^2^2p^5^), providing a consistent description of the electronic states. Within this computational framework, the equilibrium structural parameters, formation energies, band structures, density of states (DOS/PDOS), elastic constants, and optical spectra, including dielectric function, absorption coefficient, reflectivity, refractive index, and optical conductivity, were systematically analyzed. The elastic stiffness constants (*C*_*ij*_) were obtained using the finite-difference stress–strain method implemented in Python (version 3.0) *via* Google Colab scripts. X-ray diffraction (XRD) simulations and molecular dynamics (MD) calculations were carried out using the Forcite module, with MD simulations performed in the NVT ensemble at 300 K, 600 K, and 900 K using a 1 fs time step for a total run time of 50 ps to assess thermal stability. Furthermore, the electronic charge density distribution was analyzed within CASTEP to gain deeper insights into chemical bonding and charge transfer characteristics of the compounds.

### 2.2 Device simulation using SCAPS-1D

The device-level simulations were performed using the Solar Cell Capacitance Simulator (SCAPS-1D), developed at the University of Gent.^32,33^ SCAPS-1D is a widely used numerical simulation tool for calculating and modeling device-level properties of thin-film solar cells, including emerging lead-free double perovskites such as A_2_AgRhF_6_ (A = K, Rb, Cs). In SCAPS-1D, the photovoltaic device is modeled as a one-dimensional multilayer stack (*e.g.*, ETL/absorber/HTL), where charge transport and electrostatics are described by solving the Poisson equation together with the electron and hole continuity equations self-consistently under illumination and applied bias to obtain the current–voltage (*J*–*V*) characteristics.^34^ The electrostatic potential *ψ*(*x*) is determined from the Poisson equation,1
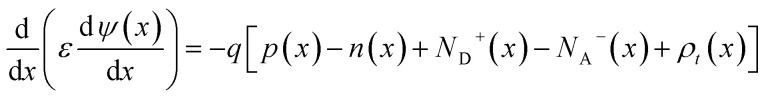
where *ε* is the dielectric permittivity, *q* is the elementary charge, *n* and *p* are the electron and hole concentrations, *N*_D_^+^ and *N*_A_^−^ are the ionized donor and acceptor densities, and *ρ*_*t*_ represents a trapped charge due to defects. The steady-state continuity equations for electrons and holes govern carrier transport,2
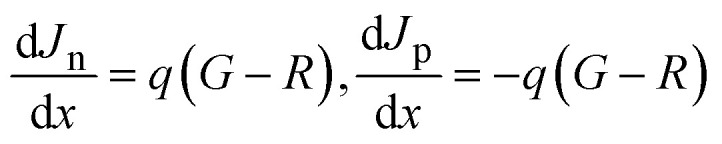
where *G* and *R* are the generation and recombination rates, respectively. The electron and hole current densities are expressed using the drift-diffusion formalism as3
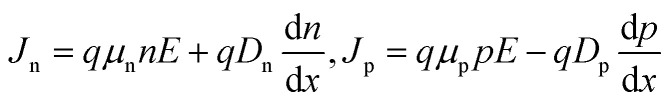
with *µ*_n_ and *µ*_p_ denoting carrier mobilities, *D*_n_ and *D*_p_ the diffusion coefficients, and 
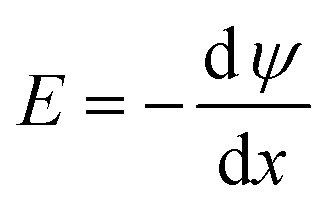
 the electric field. By simultaneously solving these coupled equations across the device layers with appropriate boundary conditions, SCAPS-1D accurately predicts the *J*–*V* characteristics, recombination losses, and overall photovoltaic performance of the simulated solar cell.

The simulated device structure consists of Au/A_2_AgRhF_6_/SnS_2_/FTO/Glass configuration, where A_2_AgRhF_6_ acts as the absorber layer and SnS_2_ serves as the electron transport layer (ETL). Au (work function is 5.35 eV) is used as the back contact, while FTO acts as the transparent conductive front contact. Material parameters of the absorber layer, including band gap, dielectric constant, and carrier mobilities were obtained from the DFT calculations and used as input parameters. Interface defect states and bulk defect densities were incorporated to analyze recombination losses and realistic device behavior. The current–voltage (*J*–*V*) characteristics were simulated under standard AM1.5G solar illumination (100 mW cm^−2^) at 300 K. From the *J*–*V* curve, the short-circuit current density (*J*_SC_), open-circuit voltage (*V*_OC_), fill factor (FF), and power conversion efficiency (PCE) were extracted. Parametric studies were performed by varying absorber thickness, defect density, doping concentration, and operating temperature to determine the optimized device configuration and to understand the influence of material properties on photovoltaic performance. The external quantum efficiency (EQE) and recombination mechanisms across the device layers were also analyzed to evaluate carrier collection efficiency and optical response. This combined DFT-SCAPS approach enables a direct correlation between intrinsic material properties and device-level photovoltaic performance.

## 3 Analysis and discussion of results using CASTEP

### 3.1 Crystal structure optimization

**Fig. 1:**
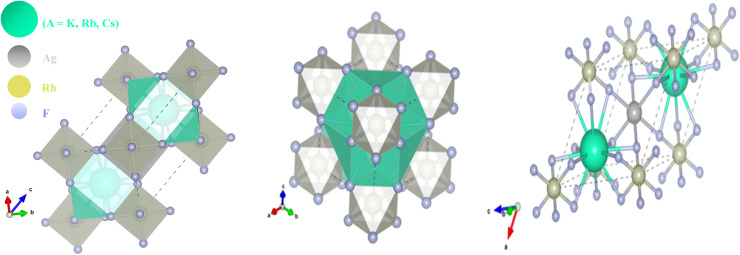
The crystal structure of A_2_AgRhF_6_ perovskites.

The double halide compound A_2_AgRhF_6_ crystallizes in the cubic perovskite-derived structure. The crystal structure is shown in Fig. 1. In this structure, Rh^3+^ ions occupy the 4a Wyckoff position (0, 0, 0) and are coordinated by six fluorine atoms to form RhF_6_ octahedra, while Ag^1+^ ions reside at the 4b site (1/2, 0, 0), forming AgF_6_ octahedra. These octahedra are corner-shared in an ordered arrangement without any octahedral tilting, maintaining the ideal cubic symmetry. The alkali metal A^1+^ ions are located at the 8c Wyckoff position (1/4, 3/4, 3/4) and are twelve-fold coordinated by fluorine atoms, forming AF_12_ cuboctahedra that share corners and faces with neighboring cuboctahedra as well as with RhF_6_ and AgF_6_ octahedra. The F^1−^ ions occupy the 24e Wyckoff position (^1^/_2_, 0, *z*) and act as bridging anions, connecting one Rh^3+^, one Ag^1+^, and four A^1+^ ions in a distorted linear coordination (where, *z* = 0.7313).^35,36^

The initial crystal structures were constructed and fully relaxed using the CASTEP module within Materials Studio, employing geometry optimization until the total energy and atomic forces converged below the prescribed thresholds. The resulting optimized configurations were subsequently visualized with the VESTA software to confirm atomic arrangements and coordination environments prior to further structural and electronic analyses.

### 3.2 Fundamental material properties analysis

Table 1 summarizes the fundamental material properties and thermodynamic properties of A_2_AgRhF_6_ double perovskites calculated using GGA-PBE and mGGA-RSCAN functionals. A clear monotonic increase in lattice constant is observed from 6.288 Å for K_2_AgRhF_6_ to 6.467 Å for Cs_2_AgRhF_6_, accompanied by an expansion of the unit-cell volume from 175.876 Å^3^ to 190.890 Å^3^, reflecting the increase in A-site ionic radius.

**Table 1:** The energy band gap, lattice constants, unit cell volume & formation enthalpy of A_2_AgRhF_6_ double perovskites

Compounds	GGA-PBE				mGGA-RSCAN		Formation energy, Δ*E*_f_ (eV per atom)	Nature
	Energy band gap, eV	Lattice constant *a* (Å)	Unit cell volume, *V* (Å^3^)	Density, g cm^−3^	Energy band gap, eV	Unit cell volume, *V* (Å^3^)		
K_2_AgRhF_6_	1.194	6.288	175.876	3.804	1.773	175.560	−4.467	Direct at X
Rb_2_AgRhF_6_	1.158	6.356	181.582	4.533	1.789	176.203	−4.455	Direct at X
Cs_2_AgRhF_6_	1.077	6.484	192.806	5.086	1.703	185.690	−4.439	Direct at X

Correspondingly, the density increases from 3.804 g cm^−3^ (K_2_AgRhF_6_) to 5.684 g cm^−3^ (Cs_2_AgRhF_6_), primarily due to the heavier alkali-metal cations. The electronic band gap obtained within GGA-PBE exhibits a gradual decrease from 1.194 eV for K_2_AgRhF_6_ to 1.077 eV for Cs_2_AgRhF_6_, indicating reduced crystal-field splitting and enhanced orbital delocalization with lattice expansion. In contrast, the mGGA-rSCAN functional yields larger band gaps, ranging from 1.773 eV (K_2_AgRhF_6_) to 1.701 eV (Cs_2_AgRhF_6_), while preserving the same decreasing trend. The thermodynamic stability of the double perovskites is evaluated through their formation energy, calculated using the following expression,

4 Δ*E*_f_ = *E*_tot_(A_2_AgRhX_6_) − 2*E*(A) − *E*(Ag) − *E*(Rh) − 6*E*(X)

All compounds exhibit negative formation enthalpies, confirming their thermodynamic stability, with Δ*E*_f_ varying from −4.467 eV per atom for K_2_AgRhF_6_ to −4.439 eV per atom for Cs_2_AgRhF_6_. The slightly reduced magnitude of Δ*E*_f_ for larger A-site cations suggests a marginal weakening of structural stability, consistent with lattice expansion and tolerance-factor considerations.

### 3.3 X-ray diffraction (XRD) analysis

When X-rays interact with a crystalline solid, atoms periodically arranged along lattice planes coherently scatter the incident radiation in specific directions, producing a characteristic diffraction pattern governed by Bragg's law,

5 *nλ* = 2*d* sin *θ*

where *d* denotes the interplanar spacing, *θ* represents the diffraction angle, *λ* is the wavelength of the incident X-ray, and *n* corresponds to the order of reflection.^37^ Analysis of the resulting 2*θ* peak positions and their corresponding intensities enables identification of crystalline phases, determination of lattice parameters, estimation of crystallite size, detection of structural defects, and evaluation of micro strain within the crystal lattice. The initial crystallographic structures of the investigated compounds were obtained from the Materials Project and subsequently subjected to full geometric optimization using the Materials Studio software package to achieve energetically stable configurations suitable for further analysis. Following structural relaxation, the Forcite module was employed to calculate the simulated X-ray diffraction (XRD) patterns for each material individually, ensuring consistency between the optimized atomic configurations and the predicted diffraction characteristics.

**Fig. 2:**
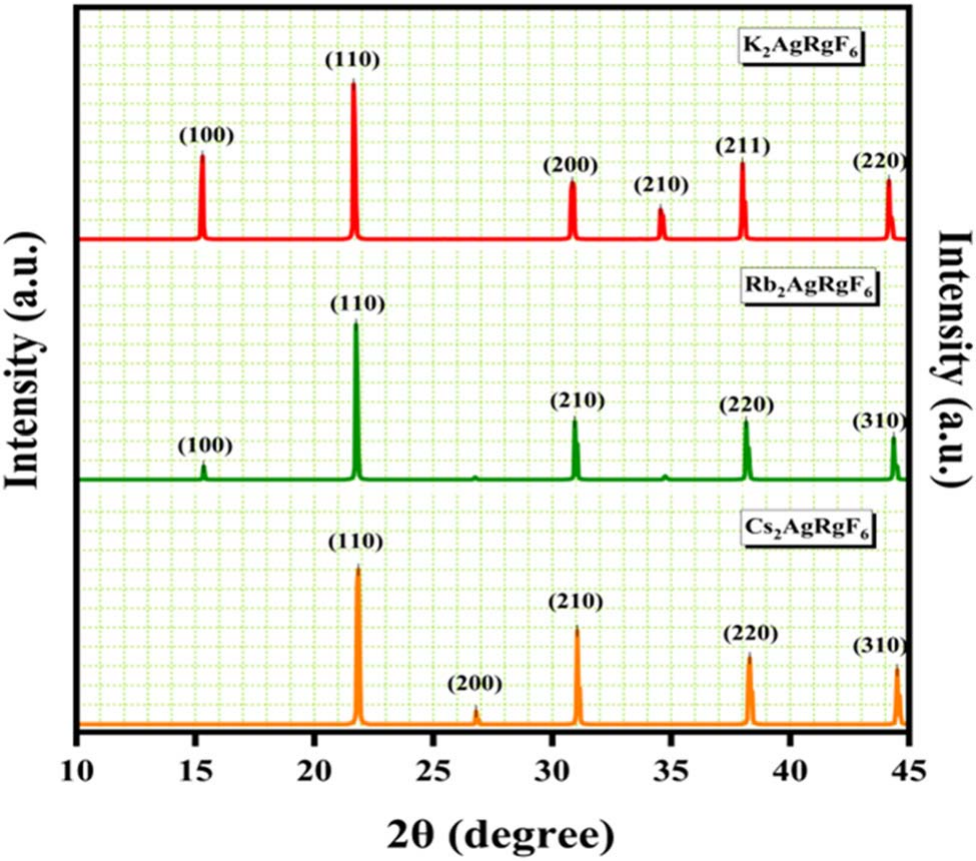
X-ray diffraction (XRD) patterns of A_2_AgRhF_6_ double perovskite compounds showing the effect of A-site cation size on crystal structure and lattice expansion.

Fig. 2 illustrates the calculated X-ray diffraction (XRD) patterns of the cubic double-perovskite fluorides A_2_AgRhF_6_ in the 2*θ* range of 10° to 45°, highlighting their structural characteristics and systematic trends. Fig. 2 shows K_2_AgRhF_6_ and Rb_2_AgRhF_6_, and Cs_2_AgRhF_6_ exhibit sharp and well-defined diffraction peaks corresponding to characteristic cubic reflections such as (100), (110), (200), (210), (211), (220), and (310), confirming that they crystallize in a stable cubic double-perovskite structure without symmetry reduction. The pronounced intensity of the (110) peak across all compositions indicates a typical ordering of Ag–Rh octahedral units. A systematic shift of diffraction peaks toward lower 2*θ* values is observed from K → Rb → Cs, reflecting the increase in A-site ionic radius and the associated lattice expansion, while preserving the overall crystal symmetry.

The absence of impurity or secondary peaks further confirms the phase purity and structural stability of these compounds, indicating that A-site substitution does not disrupt the long-range ordering of the Ag–Rh–F framework.

From an application perspective, these XRD results confirm the feasibility of A-site engineering in A_2_AgRhF_6_, enabling controlled tuning of lattice parameters without structural degradation an essential factor for band-gap and optical-property modulation. The sharp diffraction features indicate high crystallinity and low structural disorder, which are favorable for efficient charge transport and reduced carrier scattering. This makes these materials promising candidates for optoelectronic and UV-visible photonic applications. Additionally, their structural robustness supports their potential use as stable host lattices for doping and defect engineering, offering opportunities for further optimization in applications such as photocatalysis and wide-band-gap electronic devices.

### 3.4 Structural stability parameters

To predict whether a compound is stable or not, the tolerance factor (*t*) and the octahedral factor (*µ*) play a crucial role. The Table 2 presents the structural stability parameters of halide double perovskites in terms of the Goldschmidt tolerance factor (*t*) and octahedral factor (*µ*), calculated using the Goldschmidt relations.^38,39^ The Goldschmidt tolerance factor, defined as,6
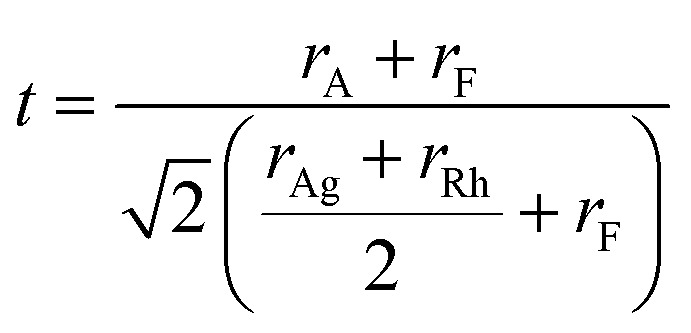


**Table 2:** Shannon ionic radii (*r*), octahedral factor (*µ*), Goldschmidt tolerance factor (*t*), and new tolerance factor (*τ*) for A_2_AgRhF_6_ (A = K, Rb, Cs) double perovskites

Ref.	Materials	*r*_A_/Å	*R*[_Ag^+^_]/Å	*R*[_Rh^3+^_]/Å	*r*_B_	*R*[_X^−^_]/Å	*t*	*µ*	*τ*
This work	K_2_AgRhF_6_	1.64	1.15	0.66	0.90	1.33	0.94	0.68	3.74
	Rb_2_AgRhF_6_	1.72	1.15	0.66	0.90	1.33	0.96	0.68	3.73
	Cs_2_AgRhF_6_	1.88	1.15	0.66	0.90	1.33	1.01	0.68	3.73
44	Li_2_AgRhCl_6_	0.92	1.15	0.67	0.91	1.81	0.71	0.50	75.10
	Na_2_AgRhCl_6_	1.39	1.15	0.67	0.91	1.81	0.83	0.50	4.59
	K_2_AgRhCl_6_	1.64	1.15	0.67	0.91	1.81	0.90	0.50	4.05
	Rb_2_AgRhCl_6_	1.72	1.15	0.67	0.91	1.81	0.92	0.50	3.96
	Cs_2_AgRhCl_6_	1.88	1.15	0.67	0.91	1.81	0.96	0.50	3.84

depends on the ionic radii of the A-site cation (*r*_A_), B-site cation (*r*_B_), and halide anion (*r*_X_), and indicates how well the ions fit into the perovskite lattice, with values between 0.8 and 1.0 typically corresponding to stable structures.

For improved prediction accuracy, the new tolerance factor (*τ*) has also been employed.^40^ It considers both ionic size mismatch and charge effects and is expressed as,7
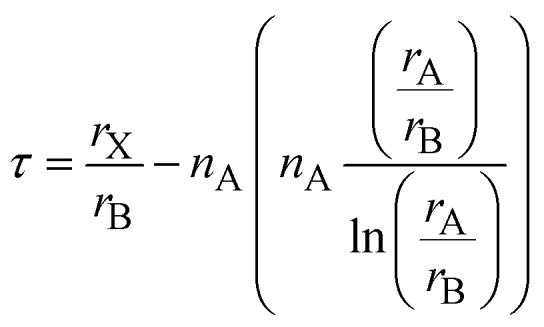


For alkali metals (A = K, Rb, Cs), the oxidation state is *n*_A_ = +1, and the expression simplifies to,8
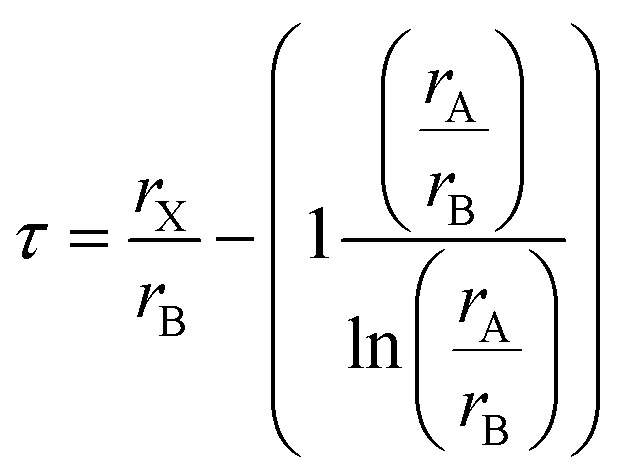


The octahedral factor (*µ*) evaluates the size compatibility of the B-site cations within the BX_6_ octahedral coordination and is defined as,^41^9
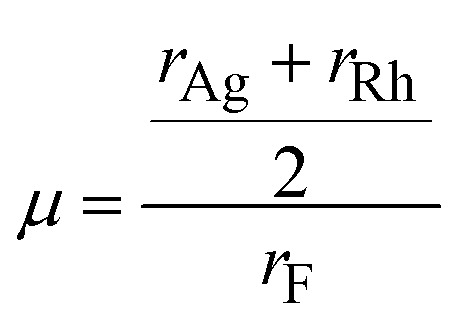


For stable perovskite formation, *µ* generally lies within the range 0.414 to 0.732.^42^ As presented in Table 2, all A_2_AgRhF_6_ compounds exhibit a constant octahedral factor of *µ* = 0.68, confirming a well-matched and stable B-site ionic environment across the series. In contrast, the tolerance factor (*t*) increases systematically with increasing A-site ionic radius, rising from 0.94 for K_2_AgRhF_6_ to 0.96 for Rb_2_AgRhF_6_ and 1.01 for Cs_2_AgRhF_6_, reflecting gradual lattice expansion due to the larger alkali-metal cations while maintaining the integrity of the Ag–Rh–F octahedral framework. Furthermore, the calculated new tolerance factors fall within the range *τ* = 3.73 to 3.79, which is below the reported stability threshold (*τ* < 4.18). These results confirm that A-site substitution among alkali metals preserves structural stability in A_2_AgRhF_6_ double perovskites according to both the classical and modified tolerance factor criteria.^43^

Accordingly, K_2_AgRhF_6_ (*t* = 0.94) and Rb_2_AgRhF_6_ (*t* = 0.96) are expected to exhibit slight octahedral tilting associated with under-sized A-site accommodation, whereas Cs_2_AgRhF_6_ (*t* = 1.01) approaches an ideal cubic perovskite geometry with minimal structural distortion. The comparison with chloride analogues (A_2_AgRhCl_6_, ref. 44) further highlights the effect of anion size, where reduced *µ* values (0.50) and lower tolerance factors (0.71 to 0.96) indicate comparatively higher structural flexibility and distortion in chloride-based systems.

### 3.5 Bonding characteristics

**Fig. 3:**
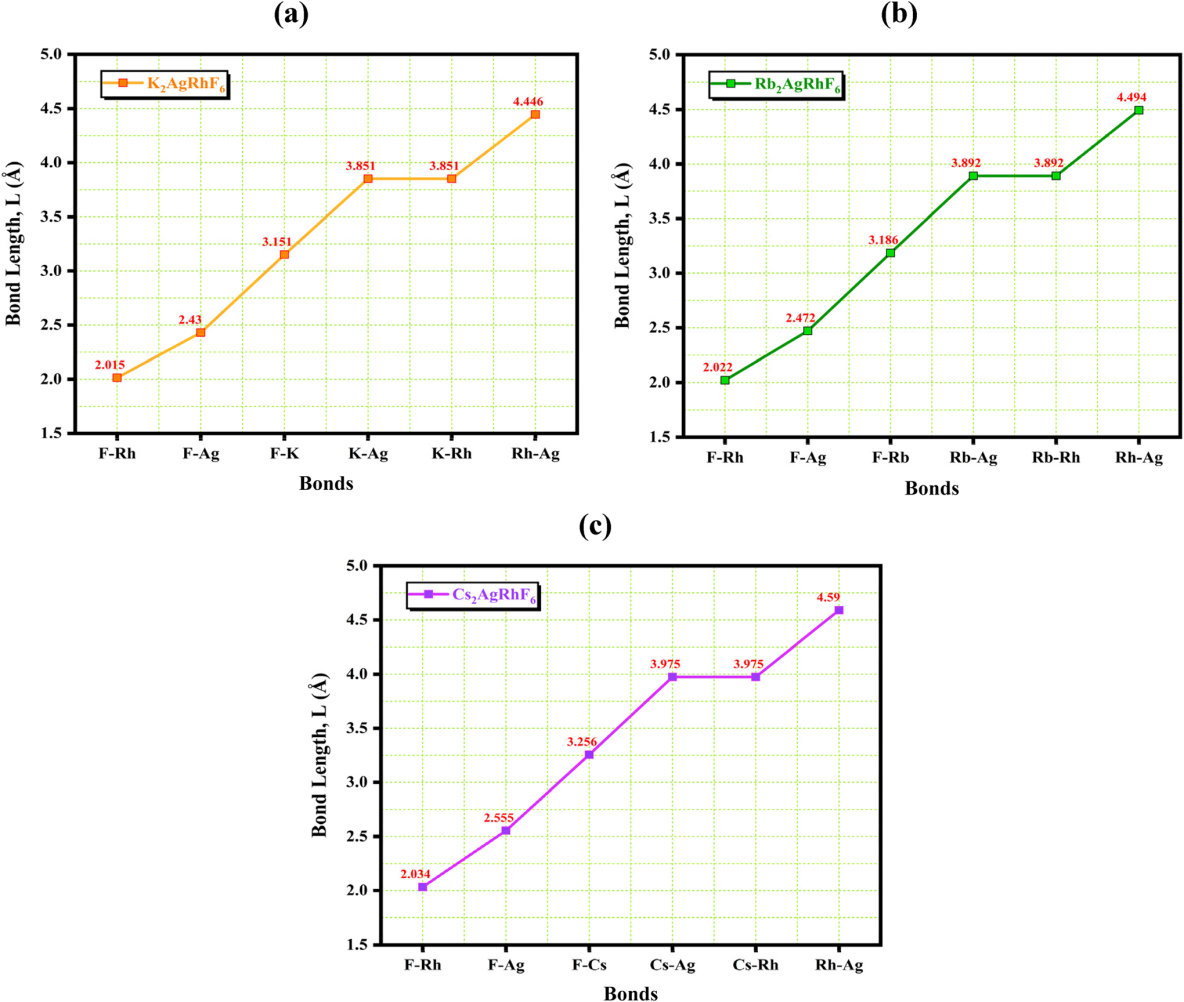
The variation of bond length within the atom of A_2_AgRhF_6_ double perovskites.

As shown in Fig. 3–(c), the calculated bond lengths exhibit a systematic variation across the A_2_AgRhF_6_ (A = K, Rb, Cs) series, reflecting changes in the local coordination environment and structural stability of these double perovskite fluorides. In Fig. 3(a), corresponding to K_2_AgRhF_6_, the Rh–F bond length is 2.015 Å, which is significantly shorter than the Ag–F bond length of 2.430 Å, indicating stronger Rh–F interactions and a more rigid RhF_6_ octahedron. The K–F distance is 3.151 Å, while the K–Ag and K–Rh separations are both 3.851 Å.

With substitution of K by the larger Rb ion, Fig. 3(b) shows that all bond lengths increase slightly. The Rh–F and Ag–F bond lengths increase to 2.022 and 2.472 Å, respectively, whereas the Rb–F distance becomes 3.186 Å. Similarly, the Rb–Ag and Rb–Rh separations increase to 3.892 Å, indicating a gradual lattice expansion and a reduction in the connectivity between adjacent AgF_6_ and RhF_6_ octahedra. A further increase in the A-site ionic radius to Cs leads to additional bond elongation, as illustrated in Fig. 3(c). In Cs_2_AgRhF_6_, the Rh–F and Ag–F bond lengths reach 2.034 and 2.555 Å, respectively, while the Cs–F distance increases to 3.256 Å. The Cs–Ag and Cs–Rh separations also increase to 3.975 Å. The systematic bond-length enlargement from Fig. 3(a)–(c) indicates progressive lattice expansion accompanied by enhanced octahedral tilting and distortion within the interconnected AgF_6_–RhF_6_ framework.

The shorter Ag–F and Rh–F bonds in Fig. 3(a) imply stronger hybridization between Ag-4d/Rh-4d and F-2p orbitals, which is expected to produce broader electronic bandwidths and stronger crystal-field effects. In contrast, the bond elongation observed in Fig. 3(b) and especially Fig. 3(c) weakens the orbital overlap, thereby favoring narrower bands and possible modifications in the electronic band gap. Although the A-site cations do not contribute directly to the states near the Fermi level, their increasing ionic size indirectly tunes the electronic structure and stability through structural expansion and octahedral distortion.

### 3.6 Electronic properties analysis

**Fig. 4:**
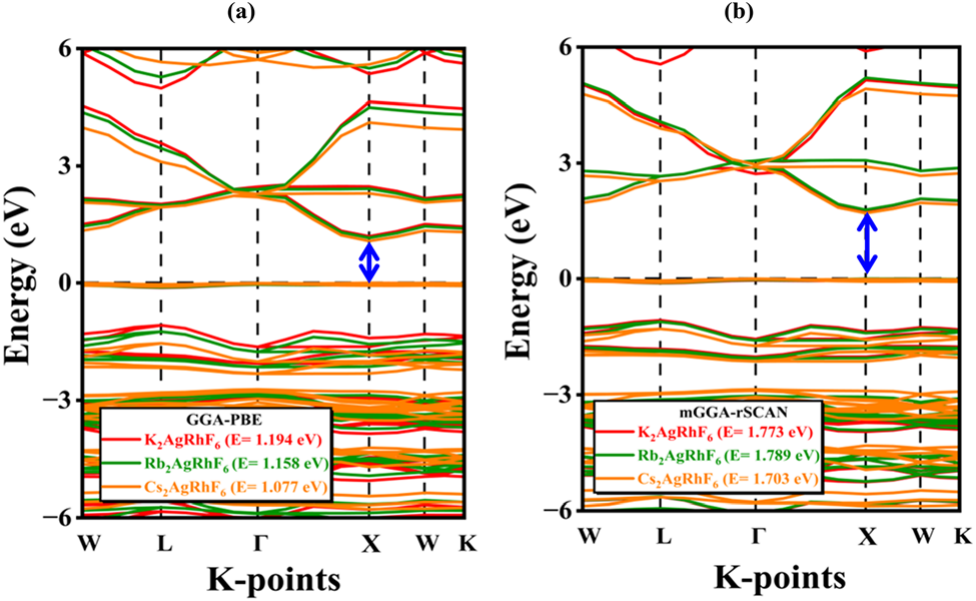
Electronic band structures of A_2_AgRhF_6_ double perovskites calculated using (a) GGA-PBE and (b) mGGA-rSCAN functionals.

The electronic band structure is crucial for identifying the semiconducting nature of these compounds. Fig. 4(a and b) presents the calculated band structures of A_2_AgRhF_6_ using both GGA-PBE and mGGA-rSCAN functionals. As expected, GGA-PBE underestimates the band gap, while mGGA-rSCAN provides improved accuracy due to its better treatment of electron correlation. The GGA-PBE functional predicts direct band gaps ranging from 1.194 to 1.077 eV (Table 1 and Fig. 4(a)), whereas the mGGA-rSCAN approach yields comparatively higher values in the range of 1.773 to 1.701 eV (Table 1 and Fig. 4(b)). Among the studied compounds, K_2_AgRhF_6_ exhibits the largest band gap, while Cs_2_AgRhF_6_ shows the smallest. The systematic trend from K → Rb → Cs reveals a gradual decrease in the band gap with increasing A-site ionic radius, which can be attributed to lattice expansion and its influence on Rh–F bond lengths and Ag–F hybridization. Orbital-resolved analysis indicates that the conduction-band minimum (CBM) is primarily derived from Rh-d states with minor Ag contributions, while the valence-band maximum (VBM) is dominated by F-p states, highlighting the critical role of Rh–F and Ag–F covalency in determining the band-edge characteristics.

Furthermore, Fig. S1(a–c) presents the electronic band structures of A_2_AgRhF_6_ (A = K, Rb, Cs) calculated with spin–orbit coupling (SOC) along the high-symmetry *k*-path (*W*–*L*–*Γ*–*X*–*W*–*K*). Upon inclusion of SOC, the materials preserve their semiconducting nature, exhibiting direct band gaps of approximately 1.118 eV for K_2_AgRhF_6_ (Fig. S1(a)), 1.089 eV for Rb_2_AgRhF_6_ (Fig. S1(b)), and 1.011 eV for Cs_2_AgRhF_6_ (Fig. S1(c)). These values are found to be consistent with the corresponding band gaps obtained without SOC, as shown in Fig. 4(a), indicating that SOC has a moderate effect on the band gap magnitude in these systems. The valence band maximum lies just below 0 eV, while the conduction band minimum appears slightly above it, confirming the presence of a finite band gap. The inclusion of SOC introduces noticeable band splitting near the Fermi level (0 eV), particularly in the upper valence region (−1 to 0 eV) and the lower conduction region (0 to 2 eV), due to spin–orbit interactions associated with the heavy Rh atom. Additionally, the deeper valence bands extend down to approximately −6 eV, showing slight redistribution and splitting. A similar direct at →X band-gap nature has also been reported in related halide double perovskites such as Rb_2_AgRhCl_6_ and Cs_2_AgRhCl_6_, as studied by Pradeep R. Varadwaj *et al.*, further supporting the consistency of electronic structure trends within this material family.^44^

Furthermore, the mGGA-rSCAN functional predicts more dispersive conduction bands compared to GGA-PBE, indicating improved carrier mobility. These results confirm that both the exchange correlation functional and the A-site cation size critically determine the electronic structures of A_2_AgRhF_6_ perovskites, offering a viable route to tune their band gaps for optoelectronic and photovoltaic applications.^45,46^

#### 3.6.1 Partial and total density of states (PDOS and TDOS) analysis

The electronic structure of the compound is further analyzed by the total density of states and partial density of states. The total number of available electronic states per unit energy and per unit volume at energy *E* and the density at which the states are distributed throughout the band structures of the entire material are represented by its TDOS, while the Partial density of states decomposes the TDOS into contribution from selected atoms, orbitals, or manifolds and reveals which chemical species and orbitals dominate specific energy ranges.^47,48^

Fig. S2 shows the total density of states (TDOS) of A_2_AgRhF_6_ (A = K, Rb, Cs) double perovskites, comparing calculations with and without spin–orbit coupling (SOC) over an energy range from −6 to 6 eV, where the vertical dashed line at 0 eV represents the Fermi level. In Fig. S2(a) K_2_AgRhF_6_, Fig. S2(b) Rb_2_AgRhF_6_, and Fig. S2(c) Cs_2_AgRhF_6_ the TDOS profiles exhibit similar overall features, confirming their semiconducting nature due to the clear gap around the Fermi level where the density of states approaches zero. The valence region, extending from approximately −6 to 0 eV, shows multiple pronounced peaks, particularly strong contributions around −5 to −3 eV and −3 to −1 eV, while the conduction region above 0 eV displays relatively smaller and broader peaks up to 6 eV. Upon inclusion of SOC (blue dashed lines), only minor modifications are observed in comparison to the non-SOC results (red solid lines), with slight shifts and smoothing of peaks, especially near the band edges around −1 to 2 eV, indicating weak but noticeable spin–orbit effects. Additionally, a gradual reduction in the band gap from K to Cs is consistent with the band structure results, reflected by the slight narrowing of the zero-DOS region around the Fermi level.

**Fig. 5:**
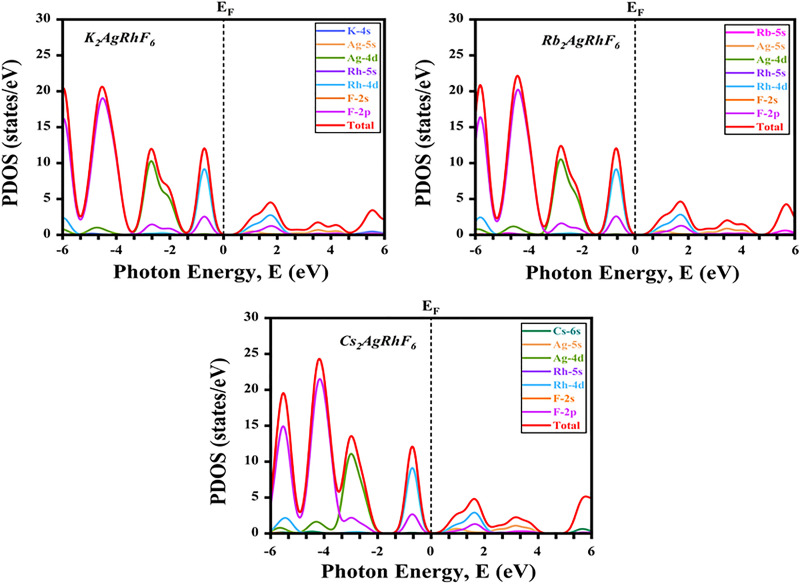
Partial density of states of A_2_AgRhF_6_ double perovskite materials.

The partial density of states (PDOS) of A_2_AgRhF_6_ provides key insight into the orbital contributions governing bond formation. As shown in Fig. 5, the valence band is dominated by fluorine and transition-metal states. The deep valence region consists primarily of F-2p orbitals, exhibiting broad and intense features several electron volts below the Fermi level (*E*_F_), while the upper valence region (−4 to 0 eV) arises mainly from Ag-4d and Rh-4d states. Notably, Rh-4d states extend closest to *E*_F_ and define the valence-band maximum (VBM), whereas Ag-4d contributes to slightly deeper features. A pronounced hybridization between F-2p and Rh-4d states is observed in the −6 to −2 eV range, indicating strong Rh–F covalent bonding, while additional Ag-4d/F-2p overlap reflects mixed covalency within the fluorine coordination environment. On the conduction side, the conduction-band minimum (CBM) is primarily governed by Rh-4d states with modest F-2p admixture, confirming the d–p antibonding nature of the lowest unoccupied bands. Higher conduction features up to 10 eV above *E*_F_ originates mainly from Rh-4d and Ag-4d antibonding states, with minor contributions from F-2p. The A-site cations contribute negligibly near the band edges; their K-4s, Rb-5s, and Cs-6s semi core states appear as weak features far from the Fermi level, indicating that these ions primarily act as electrostatic stabilizers of the perovskite lattice. Across the series (K, Rb, Cs), increasing ionic radius induces only minor shifts in semi core states and slightly modulates the degree of d–p hybridization, without altering the overall electronic structure. The PDOS analysis confirms that the band edges are dominated by Rh-4d and Ag-4d states strongly hybridized with F-2p orbitals, while A-site cations remain electronically inactive. Consequently, the band gap and electronic properties are governed almost entirely by the transition-metal fluorine framework, suggesting that A-site substitution can tune lattice parameters without introducing detrimental states near the Fermi level. These features highlight the suitability of A_2_AgRhF_6_ compounds as stable wide-gap semiconductors with robust ionic–covalent bonding characteristics.

### 3.7 Effective mass and carrier transport properties

To investigate the carrier transport characteristics of A_2_AgRhF_6_ double perovskites, particular attention was paid to the band-edge regions, as carrier mobility is primarily determined by the curvature of the conduction-band minimum (CBM) and valence-band maximum (VBM). The electron 
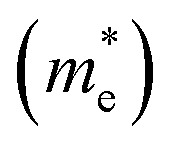
 and hole 
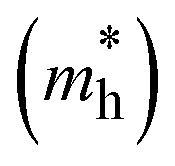
 effective masses were calculated from parabolic fitting of the band extrema according to,10
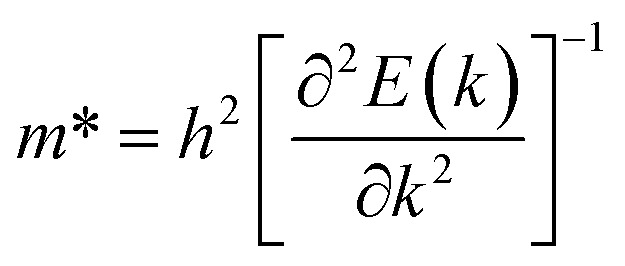


Using these effective masses, the effective density of states in the conduction and valence bands were evaluated as,11
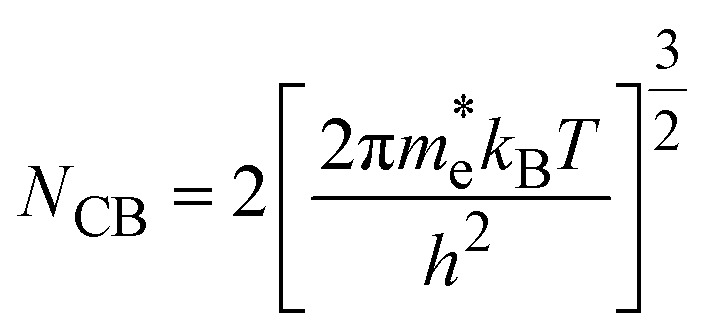
12
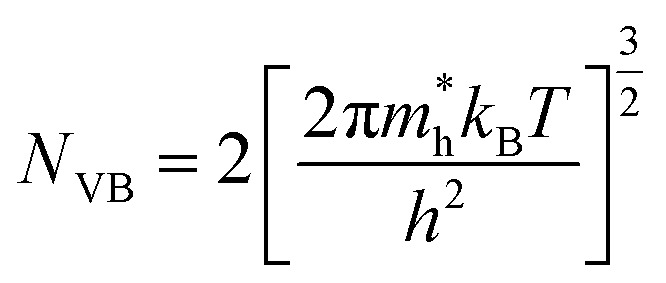


The carrier mobility was then estimated using13
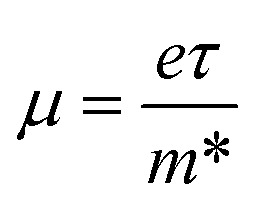


Table S1 summarizes the calculated carrier effective masses, densities of states, and mobilities of A_2_AgRhF_6_ double perovskites at 300 K, assuming a carrier relaxation time of *τ* = 10^−14^ s, where *k*_B_ is the Boltzmann constant and *e* is the elementary charge. The hole effective mass remains nearly constant across the A-site series (0.70 to 0.72 *m*_0_), leading to an almost unchanged valence-band effective density of states (1.48 to 1.53 × 10^19^ cm^−3^) and hole mobility (24 to 25 cm^2^ V^−1^ s^−1^). This consistency indicates that the valence-band dispersion is largely insensitive to A-site substitution, resulting in nearly composition-independent hole transport. In contrast, the electron effective mass decreases from 0.45 *m*_0_ (K_2_AgRhF_6_) to 0.39 *m*_0_ (Rb_2_AgRhF_6_ and Cs_2_AgRhF_6_), accompanied by a reduction in the conduction-band effective density of states from 7.576 × 1018 cm^−3^ to 6.1 to 6.3 × 10^18^ cm^−3^ and an increase in electron mobility from 39.1 to 45 cm^2^ V^−1^ s^−1^. This trend reflects enhanced conduction-band dispersion and improved electron transport with increasing A-site ionic radius.

It should be noted that the carrier mobilities reported here are estimated using the effective-mass approximation with a fixed relaxation time. While this approach captures qualitative trends across the A-site series, it has inherent limitations. The assumption of a constant *τ* neglects scattering from phonons, defects, and impurities, which can reduce mobility in real materials. Similarly, the parabolic-band approximation is valid only near the band edges; non-parabolic or anisotropic bands may introduce deviations. Temperature-dependent scattering effects are also not considered. Consequently, the reported mobilities are reliable for comparative analysis but may differ from experimental values by approximately ±20 to 50%, consistent with prior studies on halide perovskites. Nevertheless, the observed trend demonstrates that A-site substitution can effectively tune the electron transport properties in A_2_AgRhF_6_ double perovskites.

### 3.8 Optical properties

Optical properties describe the interaction of a material with electromagnetic radiation through absorption, reflection, transmission, and refraction.^49^ They are governed by the electronic structure, band gap, and symmetry, and provide key information on electronic transitions and bonding characteristics. Analysis of optical behavior is essential for evaluating materials for optoelectronic applications such as solar cells, LEDs, lasers, photodetectors, and optical devices.^50^

#### 3.8.1 Absorption coefficient (*α*)

The absorption coefficient quantifies a material's ability to absorb incident photons per unit distance^51^ and is crucial for assessing performance in energy harvesting, optoelectronic devices, and photocatalysis.^52^ In DFT, it is obtained from the complex dielectric function, with *ε*_1_(*ω*) and *ε*_2_(*ω*) representing the real and imaginary components, respectively.^53,54^14
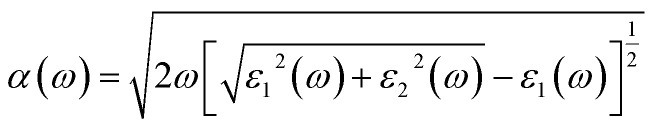


The optical absorption spectra of A_2_AgRhF_6_ (A = K, Rb, Cs) in Fig. S3(a and b) exhibit distinct energy-dependent behavior governed by their electronic structures. In the visible region (1.5 to 3.0 eV), all compounds show finite absorption, indicating partial visible-light activity despite near-infrared absorption onsets.^55^ A systematic red shift of the absorption edge is observed from K → Cs, consistent with the decreasing band gaps (1.194 → 1.077 eV), with Cs_2_AgRhF_6_ exhibiting the lowest-energy onset among the remaining compositions. Within the visible range, K_2_AgRhF_6_ shows relatively stronger absorption compared to Rb_2_AgRhF_6_ and Cs_2_AgRhF_6_, while Cs_2_AgRhF_6_ exhibits comparatively reduced intensity, reflecting variations in optical transition probabilities and orbital hybridization strength. In the ultraviolet region (>3.2 to 3.5 eV), the absorption coefficient rises sharply (>10^5^ cm^−1^) due to strong interband transitions, while additional deep-UV features (10 to 15 eV) originate from hybridized Rh-d, Ag-d, and F-p states. The wavelength-dependent spectra (Fig. S3b) further confirm strong UV absorption (*λ* < 300 nm), moderate visible response (300 to 750 nm), and negligible absorption in the infrared region (>750 nm). These characteristics highlight the potential of A_2_AgRhF_6_ compounds for UV-optoelectronic and broadband photonic applications.

#### 3.8.2 Conductivity (*σ*)

A material's optical conductivity gives a useful insight into its interaction with photons with charge carriers. It reflects the dynamic inter band electronic transitions, consisting of real and imaginary parts. In optical studies (from DFT), the frequency-dependent optical conductivity *σ*(*ω*) is complex and is written as,^56^ The complex optical conductivity is written as,^57^

15 *σ*(*ω*) = *σ*_1_(*ω*)+*iσ*_2_(*ω*)

The optical conductivity spectra comprise *σ*_1_ (real, dissipative) and *σ*_2_ (imaginary, reactive) components. As shown in Fig. S3(c), *σ*_1_ remains negligible below the absorption edge and begins to rise within the visible range (1.5 to 3.0 eV), reaching 0.2 to 0.8, indicating the onset of interband photoconductivity associated with transitions from F-p/Ag-d valence states to Rh-d conduction states. A more detailed analysis of the optical conductivity along with other optical properties (reflectivity, refractive index, dielectric function, and loss function) is presented in the SI (Fig. S3 and S4).

### 3.9 Charge density analysis

Charge density mapping provides a real-space view of electronic distribution in crystals, offering direct insight into bonding and interatomic interactions.^58^ Regions of charge accumulation and depletion distinguish bonding character: localization around electronegative atoms indicates ionic behavior, while continuous charge density between atoms reflects covalent interactions.^59^ It also reveals bonding anisotropy, orbital hybridization, and site-specific electronic contributions. For A_2_AgRhF_6_ (Fig. S5), the maps show a clear ionic-covalent interplay within the double-perovskite framework. Strong charge accumulation around F anions highlights their dominant electronegative role.^60^ Noticeable charge sharing along Rh–F and Ag–F bonds indicate partial covalency, with Rh–F exhibiting slightly stronger localization, consistent with more pronounced d–p hybridization. In contrast, A-site cations (K, Rb, Cs) are associated with low charge density, confirming their largely ionic character and negligible involvement in bonding. Across the series, the overall charge distribution remains nearly unchanged, indicating that A-site substitution primarily affects lattice dimensions and electrostatics rather than the intrinsic bonding within the AgRhF_6_ framework.

### 3.10 Elastic constants analysis

Mechanical properties describe a material's response to stress, strain, and pressure, typically characterized by elastic constants, bulk modulus, shear modulus, Young's modulus, Poisson's ratio, and hardness, providing insight into its strength, stiffness, ductility, brittleness, and elastic stability.^61^ Assessing these properties ensures a compound can withstand fabrication and operational stresses while revealing lattice stability,^62^ bonding anisotropy, and long-term durability, critical for optoelectronic devices, coatings, sensors, and structural components.^63^ The elastic stability of cubic A_2_AgRhF_6_ (A = K, Rb, Cs) double perovskites was confirmed *via* the Born criteria in eqn (S7),^64^ with all compounds satisfying these conditions. As shown in Fig. S6, K_2_AgRhF_6_ shows strong resistance to tetragonal shear (*C*_11_ − *C*_12_ = 40.455 GPa), Rb_2_AgRhF_6_ exhibits the highest hydrostatic rigidity (*C*_11_ + 2*C*_12_ = 122.897 GPa), and Cs_2_AgRhF_6_ displays moderate stiffness. Overall, lattice rigidity decreases with increasing A-site ionic size (Rb_2_AgRhF_6_ > K_2_AgRhF_6_ > Cs_2_AgRhF_6_), indicating that heavier alkali substitution slightly weakens bonding while maintaining mechanical stability, a key factor for reliable optoelectronic applications.

### 3.11 Mechanical properties and hardness

The mechanical properties of the A_2_AgRhF_6_ series reveal distinct trends influenced by the A-site cation. The bulk modulus (*B*), reflecting resistance to volumetric compression and calculated using eqn (S8),^61^ is highest for Rb_2_AgRhF_6_ (40.97 GPa), indicating a rigid lattice with strong interatomic bonding, while K_2_AgRhF_6_ (14.079 GPa) is the softest and most compressible; Cs_2_AgRhF_6_ (17.664 GPa) exhibits intermediate compressibility, as shown in Fig. S7(a). Similarly, the shear modulus (*G*_H_), representing resistance to shape deformation and obtained from Hill's average of Voigt and Reuss bounds (eqn (S9)),^65^ is greatest for Rb_2_AgRhF_6_ (13.204 GPa) and lowest for K_2_AgRhF_6_ (7.805 GPa), with Cs_2_AgRhF_6_ (9.994 GPa) showing moderate rigidity (Fig. S7(b)). Young's modulus (*Y*), measuring stiffness under uniaxial stress and calculated using eqn (S10),^65^ follows the same trend, with Rb_2_AgRhF_6_ showing the highest stiffness (35.77 GPa), Cs_2_AgRhF_6_ moderate stiffness (25.226 GPa), and K_2_AgRhF_6_ the lowest (19.765 GPa), as illustrated in Fig. S4(c). Poisson's ratio (*ν*, eqn (S11)) and Pugh's ratio (*B*/*G*, eqn (S12))^66^ indicate ductility, with Rb_2_AgRhF_6_ (*ν* = 0.354, *B*/*G* = 3.103) being ductile, K_2_AgRhF_6_ (*ν* = 0.266, *B*/*G* = 1.804) marginally ductile, and Cs_2_AgRhF_6_ (*ν* = 0.261, *B*/*G* = 1.767) near the brittle–ductile boundary (Fig. S7(d) and (e)). Hardness, reflecting resistance to permanent deformation and estimated using Tian's formula (eqn (S13)),^61,67^ is highest for K_2_AgRhF_6_ (3.331 GPa), followed by Cs_2_AgRhF_6_ (2.938 GPa) and Rb_2_AgRhF_6_ (1.660 GPa) (Fig. S7(f)), while the machinability index (eqn (S14))^68^ shows K_2_AgRhF_6_ (3.91) is easiest to process and Cs_2_AgRhF_6_ (1.214) the least (Fig. S7(g)). Average sound velocity, linked to lattice dynamics and thermal transport,^69–72^ is maximal in Rb_2_AgRhF_6_ (1911.69 m s^−1^) and lowest in K_2_AgRhF_6_ (Fig. S7(h)). Collectively, Rb_2_AgRhF_6_ emerges as the most mechanically robust compound, combining high stiffness, shear resistance, ductility, and sound velocity, making it suitable for load-bearing and pressure-resistant applications. K_2_AgRhF_6_ stands out for hardness and machinability, ideal for wear-resistant and easily processed components, while Cs_2_AgRhF_6_ exhibits moderate stiffness with near-brittle behavior, limiting its use to low-stress or protective applications.

### 3.12 Anisotropy analysis

Anisotropy analysis reveals the directional dependence of a material's physical properties, which influences mechanical, thermal, and optical behavior.^73,74^ The Zener anisotropy factor (eqn (S15)),^73^ universal anisotropy index (*A*^U^, eqn (S16)), equivalent Zener measure (eqn (S17)), and shear anisotropy factor (*A*^G^, eqn (S18)) were calculated to assess elastic anisotropy in the A_2_AgRhF_6_ series. Among the studied compounds, K_2_AgRhF_6_ shows moderate anisotropy (*A* = 0.313, *A*^U^ = 4.558, *A*^eq^ = 5.62, *A*^G^ = 0.313), Cs_2_AgRhF_6_ is nearly isotropic (*A* = 0.102, *A*^U^ = 1.135, *A*^eq^ = 2.554, *A*^G^ = 0.102), and Rb_2_AgRhF_6_ approaches quasi-isotropic behavior (*A* = 0.028, *A*^U^ = 0.285, *A*^eq^ = 1.62, *A*^G^ = 0.028) (Fig. S7). These results indicate that K_2_AgRhF_6_ may be suitable for direction-sensitive applications, whereas Rb_2_AgRhF_6_ and Cs_2_AgRhF_6_ offer more uniform mechanical performance.

### 3.13 Elastic tensor analysis (ELATE)

Fig. S9 provides a comprehensive graphical assessment of elastic anisotropy for the double perovskites K_2_AgRhF_6_, Rb_2_AgRhF_6_, and Cs_2_AgRhF_6_ using 2D polar representations in the *X*–*Y* plane and corresponding 3D surface plots of Young's modulus, shear modulus, and Poisson's ratio. This combined visualization enables a clear comparison of directional elastic behavior across the alkali-metal series. For Young's modulus (top row) in Fig. S9 and S10, all compounds exhibit noticeable anisotropy, as evidenced by the non-circular, multi-lobed shapes in the 2D polar plots. K_2_AgRhF_6_ shows a relatively pronounced four-lobed pattern with distinct maxima along specific crystallographic directions, indicating significant directional dependence of stiffness. Rb_2_AgRhF_6_ and Cs_2_AgRhF_6_, although still anisotropic, display comparatively smoother and more rounded lobes, suggesting a more uniform stiffness distribution. The 3D surface plots support these observations, where K_2_AgRhF_6_ presents more elongated lobes, while Rb_2_AgRhF_6_ and Cs_2_AgRhF_6_ form more compact and less distorted surfaces, indicating relatively reduced elastic anisotropy.

The shear modulus (middle row) in Fig. S9 and S10 demonstrates near-isotropic behavior for all compounds. The 2D polar plots for K_2_AgRhF_6_, Rb_2_AgRhF_6_, and Cs_2_AgRhF_6_ are close to circular, and the corresponding 3D surfaces resemble nearly spherical shapes, indicating minimal directional variation in resistance to shear deformation. This suggests that shear response is largely orientation-independent for these materials. Poisson's ratio (bottom row) in Fig. S9 and S10 reveals intermediate anisotropic behavior between Young's modulus and shear modulus. All compounds display four-lobed patterns in the 2D plots, indicating directional dependence of transverse strain. K_2_AgRhF_6_ and Cs_2_AgRhF_6_ exhibit relatively more pronounced lobes, suggesting larger orientation-dependent variation, whereas Rb_2_AgRhF_6_ shows comparatively reduced anisotropy with smoother contours.

### 3.14 *Ab Initio* molecular dynamic analysis (AIMD)

*Ab initio* molecular dynamics (AIMD) simulations were performed within the framework of density functional theory using the NVT ensemble with a Nosé–Hoover thermostat. A supercell of 2 × 2 × 1 was employed with a time step of 1 fs, and the total simulation time was 50 ps. The interatomic interactions were described using DFT-based forces as implemented in CASTEP, with the GGA-PBE exchange correlation functional. The system was directly initialized at the target temperatures (300, 600, and 900 K) without a prior ramping stage. Temperature control was achieved using a thermostat, and equilibration was confirmed by stable fluctuations in temperature and energy. The structure remains dynamically stable with no bond breaking or large atomic displacements throughout the simulation, confirming its robustness under thermal agitation. To evaluate the thermal stability of the A_2_AgRhF_6_ (A = K, Rb, Cs, Fr) double perovskites, AIMD simulations were performed at 300 K, 600 K, and 900 K for a total simulation time of 50 ps. The temperature fluctuations as a function of time are shown in Fig. S8(a–d). For K_2_AgRhF_6_ (Fig. S8(a)), the temperature oscillates around the target values with small fluctuations throughout the simulation, indicating stable energy exchange and no structural degradation. Even at 900 K, the system maintains equilibrium without abrupt thermal drift, demonstrating strong thermal robustness. In Rb_2_AgRhF_6_ (Fig. S8(b)), similar stable oscillatory behavior is observed. The temperature remains centered around the set values at all three temperatures, confirming dynamic equilibrium of the lattice. The relatively uniform fluctuation amplitude suggests stable atomic bonding and good thermal endurance. The Cs_2_AgRhF_6_ structure (Fig. S8(c)) also preserves thermal equilibrium during the entire 50 ps simulation. Although slightly larger fluctuations appear at elevated temperature (900 K), no systematic temperature drift occurs, indicating that the crystal framework remains intact and thermodynamically stable.

Fig. S12 illustrates the structural evolution of A_2_AgRhF_6_ (A = K, Rb, Cs) double perovskites before and after *ab initio* molecular dynamics (MD) simulations at 300 K, 600 K, and 900 K, providing insight into their thermal stability. The initial configurations (0 K) exhibit well-ordered and highly symmetric atomic arrangements, consistent with the ideal crystalline phase. At 300 K, only minor atomic displacements are observed for all compounds, and the overall lattice framework remains largely preserved, indicating good structural stability at ambient conditions. As the temperature increases to 600 K, atomic vibrations become more pronounced, leading to noticeable deviations from equilibrium positions and partial distortion of the lattice, although the fundamental framework is still retained. At 900 K, significant structural disorder is evident, characterized by substantial atomic displacements and loss of long-range order, suggesting the onset of thermal instability or possible phase transition behavior. A comparative analysis reveals a consistent trend across the series (K → Rb → Cs), where thermal-induced disorder increases with temperature; however, subtle differences in stability are observed. In particular, K_2_AgRhF_6_ maintains relatively better structural coherence, whereas Cs_2_AgRhF_6_ exhibits more pronounced distortions at elevated temperatures, likely due to the larger A-site ionic radius and the resulting lattice expansion.

### 3.15 Population analysis

How the electronic charge is distributed among the atoms in a solid is described by the population analysis of that solid. In this post DFT charge-partitioning method is utilized to quantify charge transfer, bonding character, and ionicity/covalency in a compound.^75,76^ Mulliken and Hirshfeld population analyses were employed to examine the influence of A-site substitution on the electronic charge distribution in A_2_AgRhF_6_ (A = K, Rb, Cs) perovskites. Both methods consistently indicate that the A-site alkali metals behave as strong electron donors, confirming their predominantly ionic interaction with the [AgRhF_6_]^2−^ framework. As the A-site cation evolves from K to Cs, a systematic but moderate redistribution of charge occurs, driven by the increasing ionic radius and reduced electronegativity, which subtly modifies the electrostatic potential within the lattice. Despite quantitative differences in Table S2, Mulliken charges are generally larger in magnitude than Hirshfeld charges; however, both schemes reproduce consistent qualitative trends across the series, supporting the reliability of the charge-transfer picture.^77^ The Ag and Rh centers remain positively charged in all compositions, indicating persistent electron depletion arising from Ag–F and Rh–F hybridization, with Rh exhibiting a greater degree of charge loss than Ag. Fluorine atoms consistently act as electron acceptors, maintaining the anionic character of the octahedral framework. Importantly, A-site substitution primarily tunes the extent of ionic charge donation without significantly altering the intrinsic bonding characteristics of the Ag–F and Rh–F networks. The negligible charge spilling further confirms the numerical stability and physical meaningfulness of the population analysis.^78^

## 4 SCAPS-1D device configuration and modeling parameters

The fundamental material parameters required for device simulation were obtained from density functional theory (DFT) calculations. The calculated bandgap and dielectric permittivity are presented in Tables 1 and 3, respectively. The effective density of states in the valence band (*N*_V_) and conduction band (*N*_C_), along with the electron mobility (*µ*_n_, cm^2^ V^−1^ s^−1^) and hole mobility (*µ*_h_, cm^2^ V^−1^ s^−1^), are summarized in Table S3. The electron affinity was assumed based on the calculated electronic structure and appropriate band alignment considerations. The simulated device structure consists of Au/A_2_AgRhF_6_/SnS_2_/FTO/Glass configuration, where A_2_AgRhF_6_ acts as the absorber layer and SnS_2_ serves as the electron transport layer (ETL). Au (work function is 5.35 eV) is used as the back contact, while FTO acts as the transparent conductive front contact. A simplified HTL-free device architecture is considered to evaluate the intrinsic photovoltaic performance of the absorber material. It should be noted that the absence of an HTL may lead to increased recombination losses; therefore, the results represent an idealized device configuration.

**Table 3:** Dielectric permittivity parameter in solar-cell device

Parameters	K_2_AgRhF_6_	Rb_2_AgRhF_6_	Cs_2_AgRhF_6_
Dielectric permittivity	3.103	3.088	3.132

All input parameters used for the device-level simulations are listed in Table S3. The absorber layer thickness, total defect density, and doping concentration were systematically optimized in this study to achieve enhanced photovoltaic performance. Additionally, the material parameters for the FTO and SnS_2_ layers were taken from established literature to maintain consistency with experimentally validated data.

The interface defect densities adopted in the simulation are provided in Table 4, which were selected based on previously reported studies to ensure realistic interfacial modeling. In this work, charge recombination is primarily governed by the Shockley–Read–Hall (SRH) mechanism, which is controlled by defect states present at the interfaces and within the bulk absorber. For all SnS_2_/absorber interfaces, the electron and hole capture cross sections are set to a low value of 1 × 10^−19^ cm^2^, indicating a reduced probability of carriers being captured by interface defect states and thereby favoring efficient charge extraction across the junction. The interface defects are modeled as electrically neutral, meaning they do not introduce additional fixed charges that could distort the local electric field; instead, their influence is limited to non-radiative recombination pathways. A constant total interface defect density of 1 × 10^11^ cm^−2^ is assumed for all four interfaces, representing a relatively high-quality interface consistent with well-passivated heterostructures. Maintaining identical interface defect characteristics for SnS_2_/K_2_AgRhF_6_, SnS_2_/Rb_2_AgRhF_6_, and SnS_2_/Cs_2_AgRhF_6_ ensures that any observed variations in device performance arise predominantly from intrinsic differences in absorber properties, such as band alignment, carrier mobility, and band gap evolution induced by A-site cation substitution, rather than from extrinsic interface-related artifacts. Furthermore, the ideality factor (*n*) is not explicitly defined as an input parameter in SCAPS; instead, it is implicitly determined by the dominant recombination mechanism and defect characteristics. Under the present simulation conditions, the recombination parameters correspond to an effective ideality factor in the typical range of 1–2, consistent with reported perovskite solar cell behavior.

**Table 4:** The simulated design of SnS_2_/A_2_AgRhF_6_ (where A = K, Rb, Cs, Fr) solar cells incorporates specific interface input parameters

Interfaces	Capture cross section: electrons/holes (cm^2^)	Energy with respect to reference (eV)	Defect type	Total interface defect density (cm^−2^)
SnS_2_/K_2_AgRhF_6_	1 × 10^−19^	0.600	Neutral	1 × 10^11^
SnS_2_/Rb_2_AgRhF_6_	1 × 10^−19^	0.600	Neutral	1 × 10^11^
SnS_2_/Cs_2_AgRhF_6_	1 × 10^−19^	0.600	Neutral	1 × 10^11^

Fig. S13 illustrates the simulated energy band alignment of the FTO/SnS_2_/A_2_AgRhF_6_ (A = K, Rb, Cs, Fr) solar cell architecture obtained from SCAPS-1D under equilibrium conditions. Fig. S13(a) to (c) correspond to devices employing K_2_AgRhF_6_, Rb_2_AgRhF_6_, and Cs_2_AgRhF_6_ absorber layers, respectively, each with a thickness of 0.80 µm, while the SnS_2_ electron transport layer (0.05 µm) and FTO window layer (0.05 µm) are kept identical for all configurations. The conduction band minimum (*E*_C_), valence band maximum (*E*_v_), electron quasi-Fermi level (*F*_n_), and hole quasi-Fermi level (*F*_P_) are plotted as a function of device thickness, clearly revealing the band offsets and internal electric field distribution across the heterojunctions.^79^ A systematic reduction in the absorber band gap is observed with increasing ionic radius of the A-site cation, decreasing from 1.194 eV (K_2_AgRhF_6_) to 1.158 eV (Rb_2_AgRhF_6_), and 1.077 eV (Cs_2_AgRhF_6_). This progressive band-gap narrowing enhances absorption in the visible and near-infrared regions, thereby potentially improving photocurrent generation. The corresponding shift in band edges also slightly modifies the interfacial band offsets, influencing carrier transport dynamics. SnS_2_ is employed as the electron transport layer (ETL) due to its suitable electronic structure and transport characteristics. The conduction band of SnS_2_ forms a favorable, slightly positive conduction band offset with the absorber, which promotes efficient electron extraction while avoiding significant interfacial electron accumulation. Simultaneously, its deep valence band creates a substantial valence band offset that effectively blocks hole back-transfer from the absorber to the ETL, thereby suppressing interfacial recombination.

Moreover, the wide band gap of SnS_2_ ensures high optical transparency, minimizing parasitic absorption losses, while its intrinsic n-type conductivity and reasonable electron mobility facilitate rapid charge transport toward the FTO electrode. The band diagrams further reveal a pronounced separation between *F*_n_ and *F*_p_ within the absorber region, indicating strong carrier separation under equilibrium conditions. The built-in electric field across the SnS_2_/absorber interface enhances drift-assisted carrier collection, which is consistent with the expected trend in open-circuit voltage (*V*_OC_).

### 4.1 Thickness-dependent photovoltaic performance

The absorber layer thickness critically influences the photovoltaic performance of Na_2_AgRhF_6_, K_2_AgRhF_6_, and Cs_2_AgRhF_6_-based solar cells. The thickness was varied from 0.2 to 2.3 µm, and the corresponding variations in PCE, FF, *J*_SC_, and *V*_OC_ are presented in Fig. S14(a–d). As shown in Fig. S14(a), the PCE increases markedly with thickness up to 0.8 µm for all devices. Specifically, the PCE improves from 21.70% to 29.19% for Na_2_AgRhF_6_, 22.76% to 30.42% for K_2_AgRhF_6_, and 23.58% to 30.01% for Cs_2_AgRhF_6_. This enhancement is attributed to improved light harvesting and increased photogeneration of charge carriers resulting from the extended optical path length.^80^

Beyond an absorber thickness of 0.8 µm, only marginal improvements in device performance are observed. The maximum power conversion efficiency (PCE) occurs in the range of 1.4 to 1.7 µm, reaching approximately 30.90% for K_2_AgRhF_6_ (with Na and Cs-based devices showing comparable values around 30%). However, further increasing the thickness to 2.3 µm leads to a slight decline in PCE due to enhanced bulk recombination and increased carrier transport distance, which counteract the benefits of additional light absorption.

The fill factor (FF) in Fig. S4(a) shows a gradual increase with thickness but quickly approaches saturation beyond 0.8 µm, indicating limited further improvement in charge collection efficiency. In contrast, the short-circuit current density (*J*_SC_) Fig. S4(b) exhibits a strong dependence on thickness, increasing significantly from thin layers (0.2 µm) to 0.8 µm, where it nearly saturates (*e.g.*, 40 mA cm^−2^ for K_2_AgRhF_6_). Beyond this thickness, only minor gains in *J*_SC_ are observed in Fig. S4(c), suggesting that most incident photons are already absorbed. Conversely, the open-circuit voltage (*V*_OC_) decreases monotonically with increasing thickness (*e.g.*, from 0.91 V to 0.86 V for K_2_AgRhF_6_), primarily due to enhanced recombination losses in thicker absorber layers, which reduce quasi-Fermi level splitting. Although slightly higher PCE values are obtained at larger thicknesses in Fig. S4(d), the relative improvement beyond 0.8 µm remains negligible, while recombination losses increase and *V*_OC_ declines. At 0.8 µm, all devices achieve near-optimal performance, combining high *J*_SC_, relatively large *V*_OC_, and stabilized FF, resulting in efficiencies of approximately 29 to 30%. Therefore, 0.8 µm is identified as the optimal absorber thickness, offering the best balance between efficient light absorption, minimized recombination, and overall device performance.

### 4.2 Total defect density and doping effects

Fig. S15(a) shows the effect of absorber defect density (10^10^ to 10^18^ cm^−3^) on the photovoltaic performance of K_2_AgRhF_6_, Rb_2_AgRhF_6_, and Cs_2_AgRhF_6_-based solar cells. At low defect density (10^10^ cm^−3^), high efficiencies are achieved with PCE values of 29.80% (K_2_AgRhF_6_), 31.70% (Rb_2_AgRhF_6_), and 33.77% (Cs_2_AgRhF_6_), indicating efficient charge transport and minimal recombination losses. As defect density increases to 10^14^ cm^−3^, a slight performance decline is observed, with PCE reducing to 29.19%, 30.42%, and 30.01%, respectively, accompanied by a noticeable drop in *V*_OC_ (0.91 to 0.81 V range), while *J*_SC_ remains nearly stable. This behavior reflects the onset of defect-assisted Shockley–Read–Hall recombination, which increases non-radiative losses in the absorber layer. At high defect density (10^18^ cm^−3^), severe degradation occurs, with PCE dropping to 11.4%, 11.6%, and 11.2%, along with a strong reduction in *J*_SC_ and *V*_OC_. This is attributed to dominant SRH recombination through deep-level traps, which significantly shortens carrier lifetime and diffusion length. Consequently, carrier collection becomes inefficient in practical solar cell operation, leading to major losses in current generation, voltage output, and overall device efficiency.

Fig. S15(b) illustrates the effect of absorber doping density (10^13^ to 10^20^ cm^−3^) on the photovoltaic performance of K_2_AgRhF_6_, Rb_2_AgRhF_6_, and Cs_2_AgRhF_6_-based solar cells. At low doping (10^13^ cm^−3^), relatively lower efficiencies are obtained with PCE values of 23.74% (K_2_AgRhF_6_), 24.20% (Rb_2_AgRhF_6_), and 22.86% (Cs_2_AgRhF_6_), mainly due to lower *V*_OC_ (0.66 to 0.81 V) and FF (74 to 78%). With increasing doping to 10^16^ to 10^17^ cm^−3^, device performance improves significantly as the built-in electric field strengthens, enhancing charge separation and reducing recombination losses. At the optimal doping level (10^17^ cm^−3^), the PCE reaches 27.76% (K_2_AgRhF_6_), 28.56% (Rb_2_AgRhF_6_), and 27.61% (Cs_2_AgRhF_6_), with improved *V*_OC_ (0.71 to 0.86 V) and FF (84 to 85%), while *J*_SC_ remains nearly unchanged (37.9 to 43.1 mA cm^−2^). Although higher doping levels (10^18^ to 10^20^ cm^−3^) yield further numerical increases in PCE (up to 34%), such conditions may introduce undesirable effects in real solar cell operation, including Auger recombination, bandgap narrowing, defect-assisted tunneling, and reduced carrier lifetime. These mechanisms can degrade material quality and long-term device stability. Therefore, 10^17^ cm^−3^ is identified as the optimal doping density, providing a balanced trade-off between efficient charge separation, minimized recombination, and realistic device reliability.

### 4.3 Effect of operating temperature on photovoltaic performance

The influence of operating temperature (285 to 450 K) on the photovoltaic performance of Na_2_AgRhF_6_, K_2_AgRhF_6_, and Cs_2_AgRhF_6_-based solar cells is summarized in Fig. S16. A clear degradation in device performance is observed with increasing temperature for all compositions. At 285 K, the devices exhibit maximum efficiencies of 29.67% (K_2_AgRhF_6_), 31.06% (Rb_2_AgRhF_6_), and 30.89% (Cs_2_AgRhF_6_), with high *V*_OC_ (0.79 to 0.93 V) and FF (84 to 87%), while *J*_SC_ remains nearly constant (37.9 to 43.1 mA cm^−2^). As temperature increases to 300 to 375 K, a gradual decline in PCE is observed (down to 25 to 26%), mainly driven by the reduction in *V*_OC_. At the highest temperature (450 K), the efficiencies significantly decrease to 21.83%, 22.23%, and 20.73%, accompanied by a strong drop in *V*_OC_ and FF, while *J*_SC_ remains almost unchanged. This degradation is primarily attributed to the temperature-induced increase in intrinsic carrier concentration, which enhances the reverse saturation current (*J*_0_) and reduces *V*_OC_. In addition, elevated temperatures strengthen phonon-assisted scattering and non-radiative recombination, lowering FF. Since photogeneration is governed by optical absorption, *J*_SC_ remains largely unaffected.

### 4.4 Current density–voltage and quantum efficiency analysis

Fig. S17 presents the simulated current density–voltage (*J*–*V*) characteristics under AM 1.5G illumination and the corresponding external quantum efficiency (QE) spectra of A_2_AgRhF_6_ (A = K, Rb, Cs) double perovskite solar cells at optimized device parameters. As shown in Fig. S17(a), all devices exhibit typical rectifying diode behavior with a pronounced photocurrent plateau and a sharp current drop near the open-circuit voltage, confirming efficient photogeneration and carrier extraction. At zero bias, the short-circuit current densities (*J*_SC_) are 37.88295 mA cm^−2^ (K_2_AgRhF_6_), 40.15089 mA cm^−2^ (Rb_2_AgRhF_6_), and 43.14027 mA cm^−2^ (Cs_2_AgRhF_6_), with corresponding *V*_OC_ values of 0.91175, 0.88669, and 0.80958 V. The relatively higher *V*_OC_ in K_2_AgRhF_6_ indicates reduced recombination losses and stronger quasi-Fermi level splitting. The nearly rectangular shape of the *J*–*V* curves reflects high fill factor values (>84%), demonstrating efficient charge transport, strong built-in electric field formation, and minimal resistive losses.

The QE spectra in Fig. S17(b) show strong photo-response across the visible range (300 to 900 nm), with nearly unity quantum efficiency, indicating effective photon-to-electron conversion. The absorption cut-off wavelengths extend from 1040 nm (K_2_AgRhF_6_) to 1180 nm (Cs_2_AgRhF_6_), consistent with a progressive bandgap reduction across the series. This extension enhances long-wavelength photon harvesting and leads to improved current generation, particularly for Cs_2_AgRhF_6_. To validate the reliability of the simulation methodology, a benchmark lead-free double perovskite absorber (Cs_2_NaInI_6_) was simulated under identical device conditions. The calculated photovoltaic parameters (*V*_OC_ = 1.106 V, *J*_SC_ = 20.894 mA cm^−2^, FF = 84.93%, and PCE is 19.62%) show good agreement with previously reported values (*V*_OC_ is 1.194 V, *J*_SC_ is 21.234 mA cm^−2^, FF = 89.19%, and PCE = 22.60%),^81^ as summarized in Table 5. The slight deviations can be attributed to differences in device configuration, interface properties, and simulation conditions. This consistency confirms the validity and robustness of the present SCAPS simulation approach. Furthermore, the A_2_AgRhF_6_-based devices exhibit significantly higher current density and overall efficiency compared to the benchmark system, highlighting their strong potential for future high-performance photovoltaic applications.

**Table 5:** Validation and comparison of photovoltaic performance

Material	*V*_OC_ (V)	*J*_SC_ (mA cm^−2^)	FF (%)	PCE (%)
Cs_2_NaInI_6_ (benchmark)	1.106	20.894	84.93	19.62
Cs_2_NaInI_6_ (ref. 81)	1.194	21.234	89.19	22.60
K_2_AgRhF_6_	0.912	37.883	84.52	29.19
Rb_2_AgRhF_6_	0.887	40.151	85.45	30.42
Cs_2_AgRhF_6_	0.810	43.140	85.91	30.01

### 4.5 Photovoltaic efficiency and device performance

**Fig. 6:**
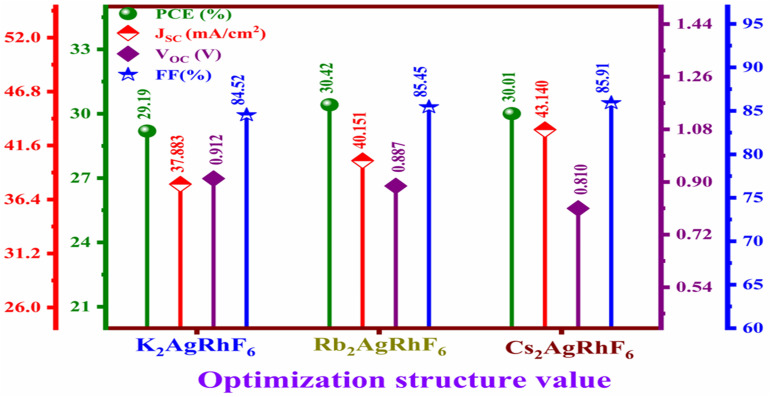
Optimized photovoltaic parameters (PCE, *J*_SC_, *V*_OC_, and FF) of A_2_AgRhF_6_ (A = K, Rb, Cs) double perovskite solar cells under optimized device conditions.

Fig. 6 presents the optimized photovoltaic parameters of A_2_AgRhF_6_ (A = K, Rb, Cs) double perovskite solar cells under optimized device conditions (absorber thickness of 0.8 µm, defect density ≤10^14^ cm^−3^, and doping density of 10^17^ cm^−3^). Fig. 6 comparatively summarizes the PCE, *J*_SC_, *V*_OC_, and FF for all compositions. Under optimized conditions, K_2_AgRhF_6_ exhibits a PCE of 29.19%, with *J*_SC_ of 37.88 mA cm^−2^, *V*_OC_ of 0.912 V, and FF of 84.52%. Rb_2_AgRhF_6_ achieves the highest efficiency of 30.42%, with *J*_SC_ of 40.15 mA cm^−2^, *V*_OC_ of 0.887 V, and FF of 85.45%. Similarly, Cs_2_AgRhF_6_ delivers a PCE of 30.01%, with *J*_SC_ of 43.14 mA cm^−2^, *V*_OC_ of 0.810 V, and FF of 85.91%. As shown in Fig. 6, *J*_SC_ increases progressively from K_2_AgRhF_6_ to Cs_2_AgRhF_6_ (37.88 → 43.14 mA cm^−2^), attributed to bandgap narrowing and enhanced long-wavelength absorption. In contrast, *V*_OC_ decreases systematically (0.912 → 0.810 V), reflecting the inverse relationship between bandgap energy and voltage, along with increased recombination in narrower-bandgap compositions. The fill factor remains consistently high (84 to 86%) across all compositions, indicating efficient charge transport and minimal resistive losses. Among the investigated absorbers, Rb_2_AgRhF_6_ demonstrates the best overall performance due to a balanced combination of current density and voltage. Therefore, K-, Rb-, and Cs-based compositions represent more feasible and practical candidates for photovoltaic development.

## 5 Cost-effectiveness and fabrication feasibility

The cost-effectiveness and practical feasibility of A_2_AgRhF_6_ (A = K, Rb, Cs) solar cells are governed by a trade-off between material stability and economic constraints. These lead-free fluoride double perovskites exhibit excellent thermodynamic, mechanical, and thermal stability, along with suitable direct band gaps and favorable optoelectronic properties, supporting their integration into conventional thin-film photovoltaic architectures and ensuring long-term device reliability under operational conditions. However, large-scale economic viability is hindered by the high cost and limited availability of rhodium and silver, which substantially increase precursor expenses. In addition, the synthesis of fluoride-based perovskites requires controlled environments and relatively high-temperature processing, increasing fabrication complexity compared to solution-processed halide counterparts. Despite these limitations, their superior chemical robustness, resistance to degradation, and environmentally benign composition provide a compelling advantage for durable and high-performance applications. Therefore, while A_2_AgRhF_6_ compounds may not yet be competitive for low-cost, large-scale photovoltaics, they remain highly promising candidates for niche and stability-critical optoelectronic devices, with future cost reduction dependent on material substitution strategies and scalable synthesis optimization.

## 6 Conclusion

In this work, a comprehensive first-principles and device-level investigation of A_2_AgRhF_6_ (A = K, Rb, Cs) fluoride double perovskites have been carried out to evaluate their structural stability, bonding characteristics, mechanical robustness, optical response, thermal stability, and photovoltaic performance. Structural optimization confirms stable cubic phases with negative formation energies ranging from −4.467 eV per atom (K_2_AgRhF_6_) to −4.402 eV per atom (Cs_2_AgRhF_6_). The lattice constant increases monotonically from 6.288 Å (K) to 6.467 Å (Cs), reflecting systematic A-site ionic size effects. Tolerance factors (0.940 to 1.031) and a constant octahedral factor (0.680) indicate structural feasibility, while calculated elastic constants satisfy the Born stability criteria, confirming mechanical stability. Electronic band structure calculations reveal direct band-gap semiconducting behavior, with GGA-PBE band gaps decreasing from 1.194 eV (K_2_AgRhF_6_) to 1.077 eV (Cs_2_AgRhF_6_), while mGGA-rSCAN predicts larger values between 1.773 and 1.701 eV, preserving the same trend. Effective mass analysis shows a gradual decrease in electron effective mass from 0.45 *m*_0_ (K) to 0.39 *m*_0_ (Rb/Cs), resulting in enhanced electron mobility from 39.1 to 45 cm^2^ V^−1^ s^−1^, whereas hole mobility remains nearly constant (25 cm^2^ V^−1^ s^−1^). Charge density and population analyses confirm strong Rh–F and Ag–F covalent interactions, while A-site cations remain predominantly ionic, indicating that the AgRhF_6_ octahedral framework governs the electronic structure. Optical analysis demonstrates strong UV absorption exceeding 10^5^ cm^−1^, moderate visible-light response, static dielectric constants between 3.088 and 3.181, and pronounced plasmonic peaks around 6 to 7 eV, highlighting suitability for UV optoelectronic applications. *Ab initio* molecular dynamics (AIMD) simulations confirm structural stability at elevated temperatures, supporting robust thermal behavior. Device simulations under AM1.5G illumination (100 mW cm^−2^) reveal promising photovoltaic performance. Under optimized conditions (doping density 10^17^ cm^−3^), Cs_2_AgRhF_6_ achieves a PCE of 30.006%, with *J*_SC_ of 43.14 mA cm^−2^, *V*_OC_ of 0.809 V, and FF of 85.91%, representing the best balance between band gap and carrier transport among the studied compositions.

## Ethical statement

The manuscript's authors agree that there is no research involving human participants, human data or tissue, or animal subjects.

## Author contributions

Imtiaz Ahamed Apon, Rifat Rafiu, Md. Sakib Hasan, Md. Azizur Rahman: methodology, validation, software, conceptualization, investigation, formal analysis, data curation, visualization, writing – original draft, and review and editing. Syeda Sayema Sumaia, Amnah Mohammed Alsuhaibani, Moamen S. Refat, Mohamed Benghanem, S. AlFaify, Noureddine Elboughdiri: investigation, validation, software, formal analysis, data curation, writing – original draft, and review and editing.

## Conflicts of interest

The authors have no conflicts of interest.

## Supplementary Material

RA-016-D6RA01913G-s001

## Data Availability

Data will be made available on reasonable request. Supplementary information (SI): there are some figures, equations, tables, and descriptions. See DOI: https://doi.org/10.1039/d6ra01913g.
