# Supplementary material for: Exploring lead-free A2AgRhF6 fluoride double perovskites for photovoltaic applications: a first-principles and device simulation study
Source: RSC Adv. 2026 May 7;16(26):24021–39. doi: 10.1039/d6ra01913g (PMC13150995; doi:10.1039/d6ra01913g)
Supplement: RA-016-D6RA01913G-s001 [file RA-016-D6RA01913G-s001.pdf]

Supplementary Information (SI) for RSC Advances.
This journal is © The Royal Society of Chemistry 2026

Exploring Lead-Free A_2AgRhF_6 Fluoride Double Perovskites for Photovoltaic Applications: A First-Principles and Device Simulation Study

Imtiaz Ahamed Apon[1], Rifat Rafiu[2], Md. Sakib Hasan[2], Md. Azizur Rahman[3], Syeda Sayema Sumaia[2], Amnah Mohammed Alsuhaibani[4], Moamen S. Refat[5], Mohamed Benghanem[6], S. AlFaify[7], Noureddine Elboughdiri[8]

[1]Department of Electrical and Electronic Engineering, Bangladesh Army University of Science and Technology (BAUST), Saidpur-5311, Bangladesh.

[2]Department of Material Science and Engineering, Khulna University of Engineering & Technology (KUET), Khulna -9203, Bangladesh.

[3]Department of Electrical and Electronic Engineering, Begum Rokeya University, Rangpur, 5400, Bangladesh.

[4]Department of Sports Health, College of Sport Sciences & Physical Activity, Princess Nourah bint Abdulrahman University P.O. Box 84428, Riyadh 11671, Saudi Arabia.

[5]Department of Chemistry, College of Science, Taif University, P.O. Box 11099, Taif 21944, Saudi Arabia.

[6]Physics Department, Faculty of Science, Islamic University of Madinah, Madinah 42351, Saudi Arabia.

[7]Department of Physics, College of Sciences, King Khalid University, P.O. Box 960, AlQura'a, Abha 61421, Saudi Arabia

[8]Chemical Engineering Department, College of Engineering, University of Ha'il, P.O. Box 2440, 81441 Ha'il, Saudi Arabia.

Corresponding Author's Email: *azizurrahmanatik49@gmail.com (Md. Azizur Rahman)

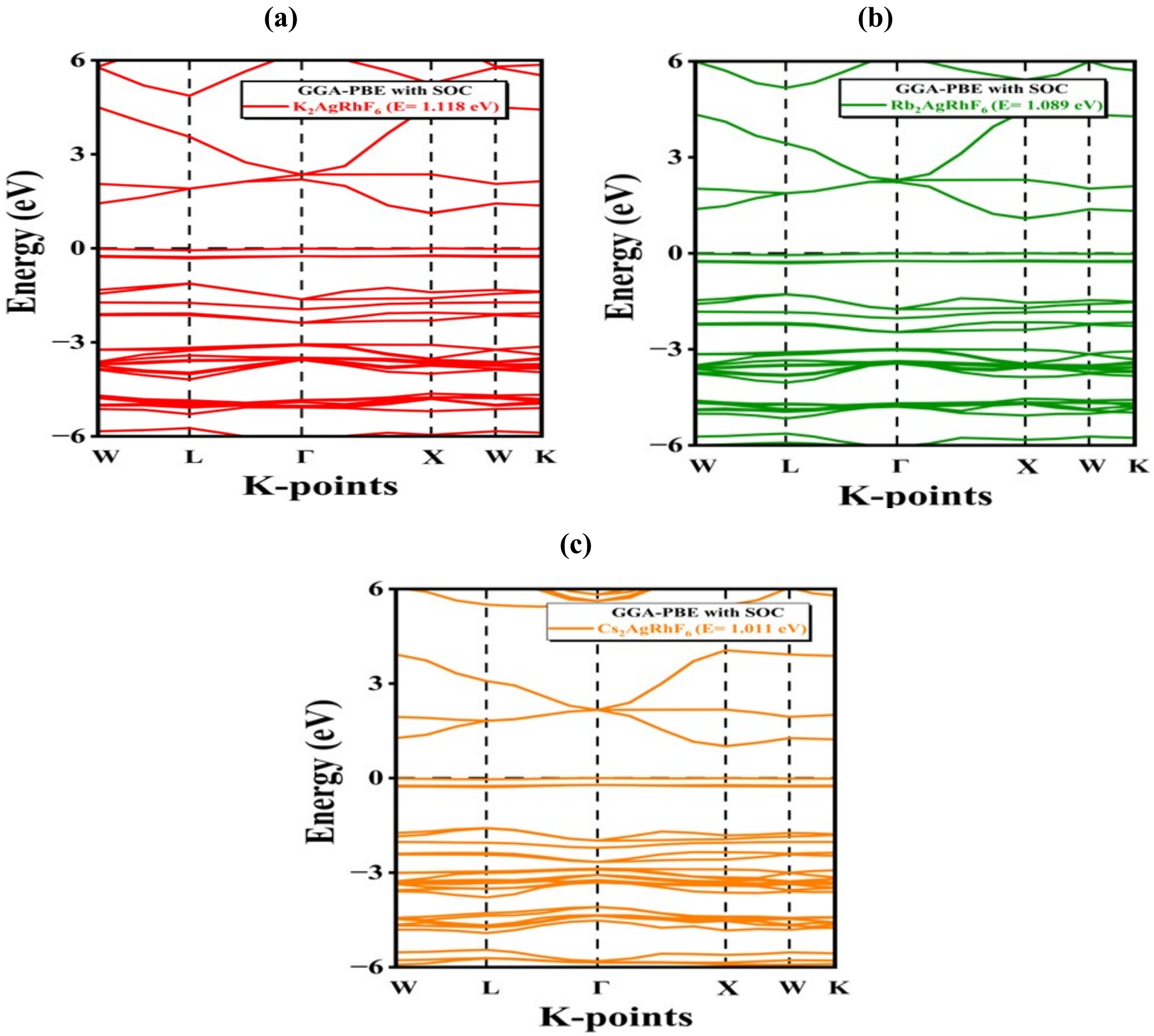

Fig. S1. Band Structure of A_2AgRhF_6 double perovskite with SOC.

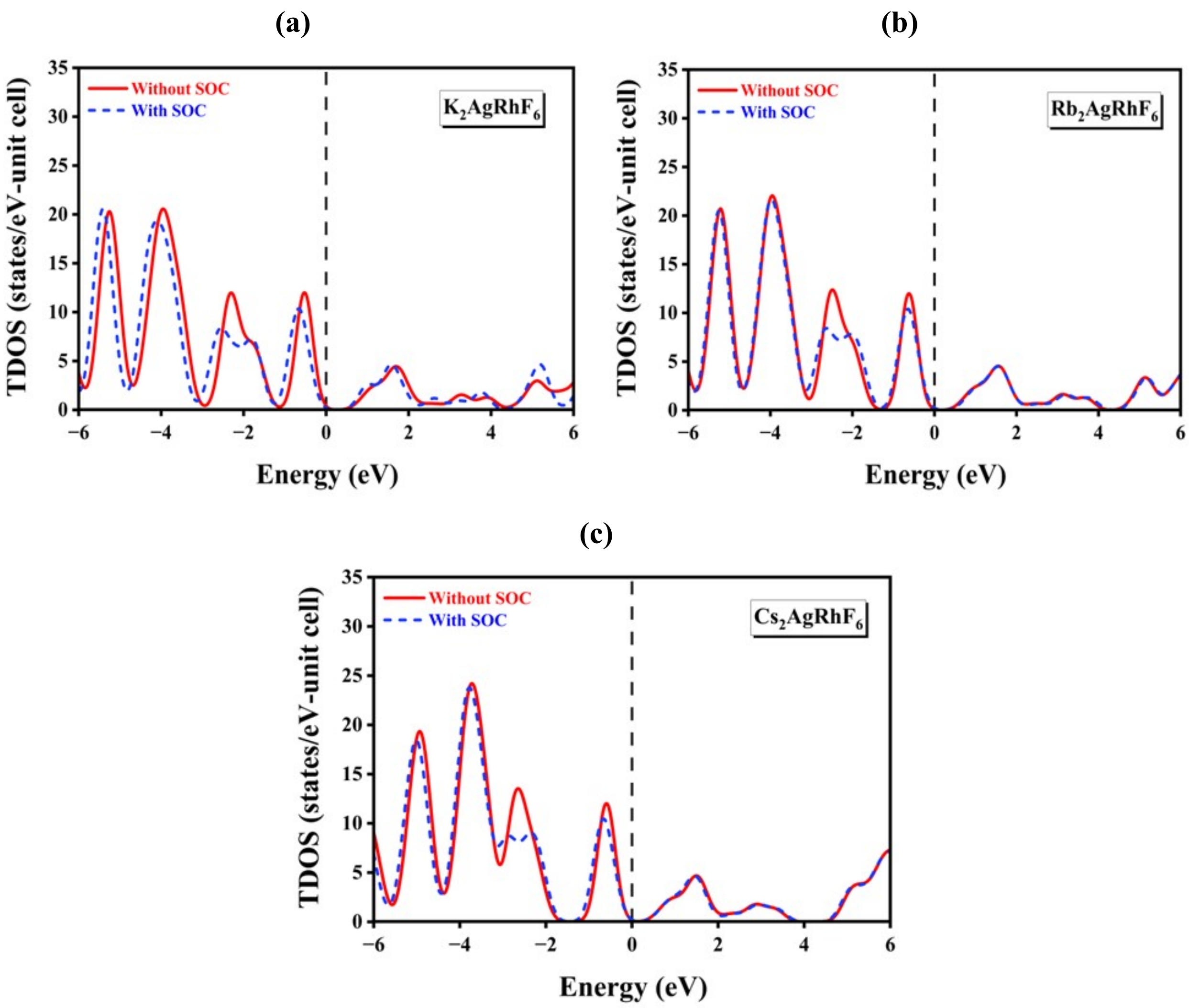

Fig. S2. Total density of states of A_2AgRhF_6 double perovskite with and without SOC.

Table S1. Carrier effective masses, densities of states, and mobilities of A_2AgRhF_6 double perovskite at 300 K.

**Materials**	**Electron effective mass, (m_e^*/m_0)**	**Hole effective mass (m_h^*/m_0)**	**C_B effective DOS, N_{CB} ($1/cm^3$)**	**V_B effective DOS, N_{VB} ($1/cm^3$)**	**Electron mobility, μ_e (cm^2/Vs)**	**Hole mobility, μ_h (cm^2/Vs)**
K_2AgRhF_6	0.45	0.71	7.576×10^{18}	1.51×10^{19}	39.1	24.7
Rb_2AgRhF_6	0.39	0.70	6.261×10^{18}	1.478×10^{19}	44.6	25.3
Cs_2AgRhF_6	0.39	0.72	6.117×10^{18}	1.532×10^{19}	45.3	24.5

1. **Optical properties:**

   1.1. **Conductivity (σ)**

A weak shoulder appears at 3 to 4 eV ($\sigma_1 \approx 0.8$ to 1.2), followed by a broader enhancement near 6 to 7 eV (1.3 to 1.8). A significant increase occurs in the deep-UV region (>10 eV), with peak σ_1 values in the 12 to 15 eV range, where Cs_2AgRhF_6 shows the highest intensity (5 to 7), while K_2AgRhF_6 and Rb_2AgRhF_6 remain lower (2 to 3). The σ_2 component exhibits oscillatory behavior with multiple zero crossings, indicating dispersive response and resonance effects near strong absorption regions [1]. In the wavelength domain (Fig. S3(d)), $\sigma_1(\lambda)$ displays sharp peaks in the UV region (<400 nm), particularly at 100 to 200 nm, corresponding to high-energy electronic transitions. Moderate conductivity persists across the visible range (400 to 550 nm) before gradually decreasing [2–4]. The $\sigma_2(\lambda)$ spectra remain dispersive with multiple zero crossings, reflecting phase variations between the incident field and induced polarization [5].

(a) **(b)**

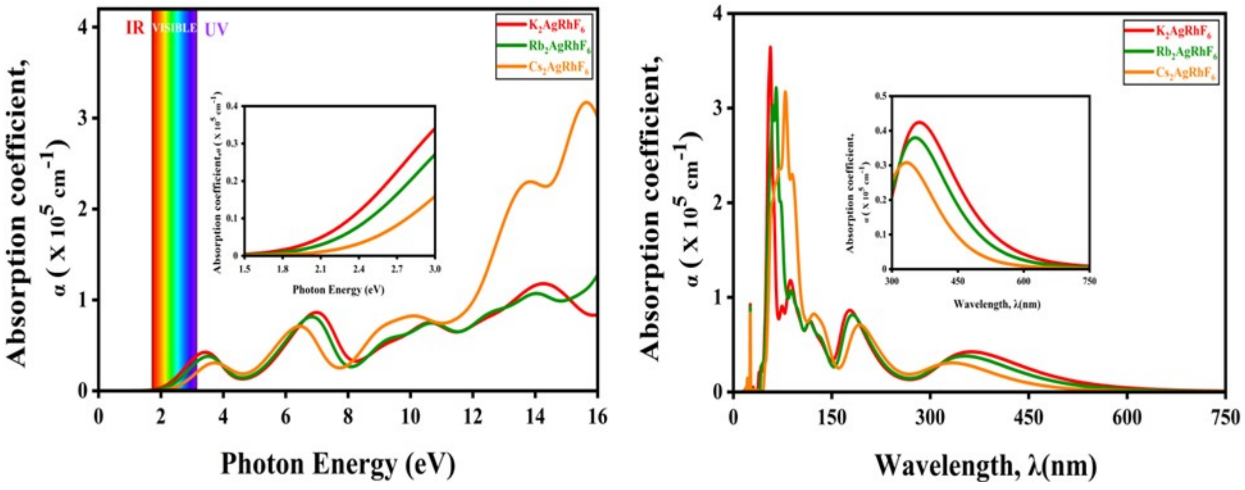

(c) **(d)**

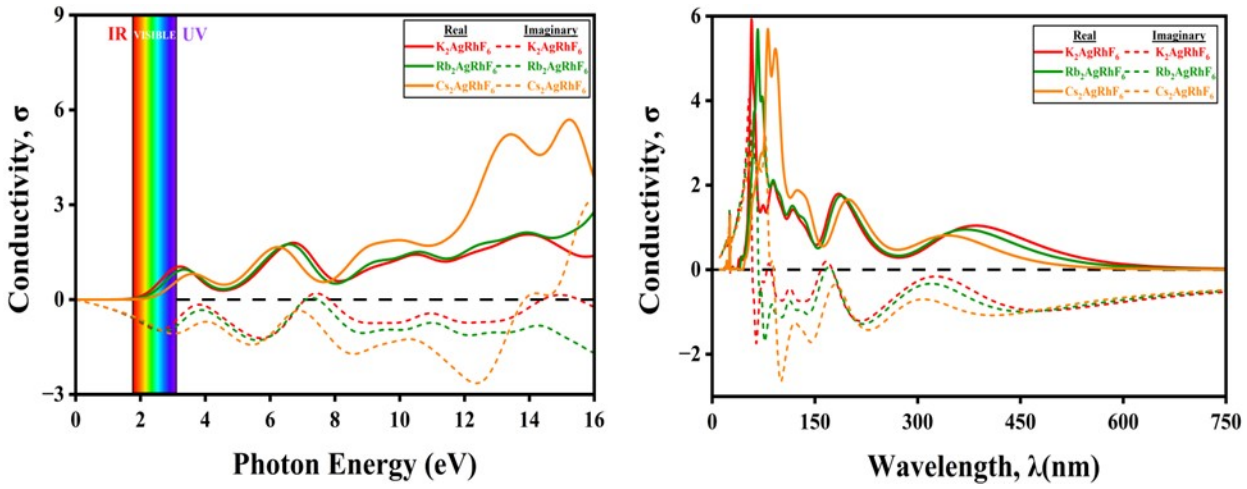

Fig. S3. Calculated (a - b) absorption coefficient and (c - d) optical conductivity spectra as a function of photon energy and wavelength of the A_2AgRhF_6 double perovskites.

1.2. Reflectivity (ρ)

Reflectivity (ρ) is an essential optical property that quantifies the fraction of incident light reflected from a material's surface, typically expressed as a value between 0 (complete absorption) and 1 (perfect reflection). It is directly related to a material's complex dielectric function, calculated using the expression below;

$$R(\omega) = \left[\frac{\left(\varepsilon(\omega)^{\frac{1}{2}} - 1\right)}{\left(\varepsilon(\omega)^{\frac{1}{2}} - 1\right)}\right]^2 \qquad \text{(S1)}$$

The reflectivity spectra of A_2AgRhF_6 double perovskites exhibit distinct features across the 0 to 16 eV photon energy range, governed by interband transitions and band-structure-related excitations. In the low-energy infrared region (0 to 2 eV), all compounds show low reflectivity (0.07 to 0.09), indicating weak reflection and strong light absorption capability. Within the visible range (1.5 to 3.0 eV), reflectivity increases moderately, reaching 0.13 to 0.16 due to the onset of interband electronic transitions. A sharp decrease around 3 to 4 eV corresponds to the plasma edge, where free-carrier effects suppress reflectivity and enhance transmission. At higher photon energies (>5 eV, UV region), the spectra display pronounced oscillations with multiple peaks and valleys, reflecting strong interband excitations. Notably, Cs_2AgRhF_6 exhibits significantly higher reflectivity (>0.2, peaking near 0.3 to 0.35 at 15 to 16 eV), whereas K_2AgRhF_6 and Rb_2AgRhF_6 remain comparatively lower (<0.15), as shown in Fig. S4(a). These

variations arise from differences in electronic structure influenced by the size and chemical nature of the alkali cations, which affect band dispersion and transition probabilities. The enhanced UV reflectivity of Cs-based compounds suggests strong photon backscattering associated with Ag-d and Rh-d interactions with F-p states.

From an application perspective, low reflectivity in the visible region supports efficient light harvesting for solar and photonic devices, while high UV reflectivity is advantageous for optical coatings, radiation shielding, and thermal management. Furthermore, the presence of a plasma edge offers tunability between transparency and reflectivity, which is critical for optoelectronic applications.

1.3. Refractive Index, (ω)

The refractive index is a key optical parameter that dictates how light propagates within a material. In CASTEP simulations, n(ω) and k(ω) are computed from the complex dielectric function, using the relation [6],

$$n(\omega) = \frac{1}{\sqrt{2}}\left[\left(\varepsilon_1(\omega)^2 + \varepsilon_2(\omega)^2\right)^{1/2} + \varepsilon_1(\omega)\right]^{1/2} \tag{S2}$$

$$k(\omega) = \frac{1}{\sqrt{2}}\left[\left(\varepsilon_1(\omega)^2 + \varepsilon_2(\omega)^2\right)^{\frac{1}{2}} - \varepsilon_1(\omega)\right]^{\frac{1}{2}} \tag{S3}$$

The refractive index spectra of A_2AgRhF_6 reveal static values (n at zero photon energy) in the range of 1.7 to 1.8, indicating moderate optical density and effective light-harvesting capability. In the infrared-to-visible region, the real part (n) shows strong dispersion, increasing to 2.0 to 2.1 near the visible range, which reflects enhanced optical confinement and supports applications in lenses, coatings, and light-guiding structures [7]. At higher photon energies, n exhibits oscillatory behavior, with minima around 3 to 4 eV and peaks reaching 2.5, as shown in Fig. S4(b). These variations correspond to interband electronic transitions that modify the dielectric response. The imaginary part (k) remains nearly zero in the infrared region, indicating negligible absorption, and increases significantly in the visible-to-UV range (2 to 6 eV), marking the onset of strong optical absorption. Prominent peaks in k coincide with intense interband transitions, confirming dominant absorption in the UV region. At higher energies, k decreases with residual oscillations, suggesting additional higher-order excitations. Collectively, the low k values in the infrared-to-visible region ensure high transparency, advantageous for solar energy harvesting, while the

elevated n enhances optical confinement for photonic applications such as waveguides, dielectric mirrors, and photonic crystals. The strong UV absorption further highlights their potential for UV photodetectors, radiation filtering, and protective optical coatings.

1.4. Dielectric function, ε(ω)

The dielectric function is a complex optical parameter that characterizes a material's response to external electromagnetic fields via polarization. It comprises a real component, representing energy storage, and an imaginary component, associated with dissipative processes such as photon absorption arising from electronic interband transitions. Eq. (S4) and (S5) describe the corresponding expressions for the dielectric function [8].

$$\varepsilon_1(\omega) = 1 + \frac{2}{\pi} P \int_0^{\infty} \frac{\omega' \varepsilon_2(\omega')}{\omega'^2 - \omega^2} d\omega' \quad \text{(S4)}$$

$$\varepsilon_2(\omega) = \frac{2\pi e^2}{\Omega \varepsilon_0} \sum_{k,v,c} \left| \langle \Psi_k^c | \hat{u}.\vec{r} | \Psi_k^v \rangle \right|^2 \delta\left(E_k^c - E_k^v - E\right) \quad \text{(S5)}$$

In Fig. S4(c), the real part of the dielectric function (ε_1) exhibits static values $\varepsilon_1(0)$ of 3.103, 3.088, and 3.132 for K_2AgRhF_6, Rb_2AgRhF_6, and Cs_2AgRhF_6, respectively, indicating a moderate-to-high polarization capability at zero frequency. These $\varepsilon_1(0)$ values are also employed as dielectric permittivity parameters in solar-cell simulations (Table 3). A relatively high static dielectric constant facilitates effective charge screening, reduces exciton binding energy, and enhances carrier mobility, highlighting the suitability of these materials for optoelectronic and energy-storage applications. In the visible region, ε_1 increases and exhibits pronounced peaks in the 2 to 3 eV range, corresponding to the onset of strong interband transitions. At higher photon energies, oscillatory behavior in ε_1 reflects successive polarization responses associated with higher-energy electronic excitations. The emergence of negative ε_1 values beyond 14 eV (notably for Cs_2AgRhF_6) signifies metallic-like behavior, attributed to plasmonic resonance and increased reflectivity [9].

The imaginary part (ε_2) exhibits distinct peaks in the UV region, particularly between 2-6 eV, consistent with strong optical absorption caused by inter-band electronic transitions from occupied F-p and Ag-d states to unoccupied Rh-d states. Multiple peaks in ε_2 across the higher energy range confirm successive optical transitions linked to the band structure. These features signify that electronic excitations dominate the optical response, directly influencing absorption,

reflectivity, and refractive index. The dielectric response has important implications for applications [10]. A high static dielectric constant enhances the suitability of these materials for energy storage and capacitor technologies. Strong dielectric activity and absorption in the UV region point toward potential in optoelectronic applications such as photodetectors, filters, and radiation shielding. Moreover, the interplay of ε_1 and ε_2 across different energy ranges suggests their usefulness in tailoring photonic devices requiring controlled transparency and reflection [11].

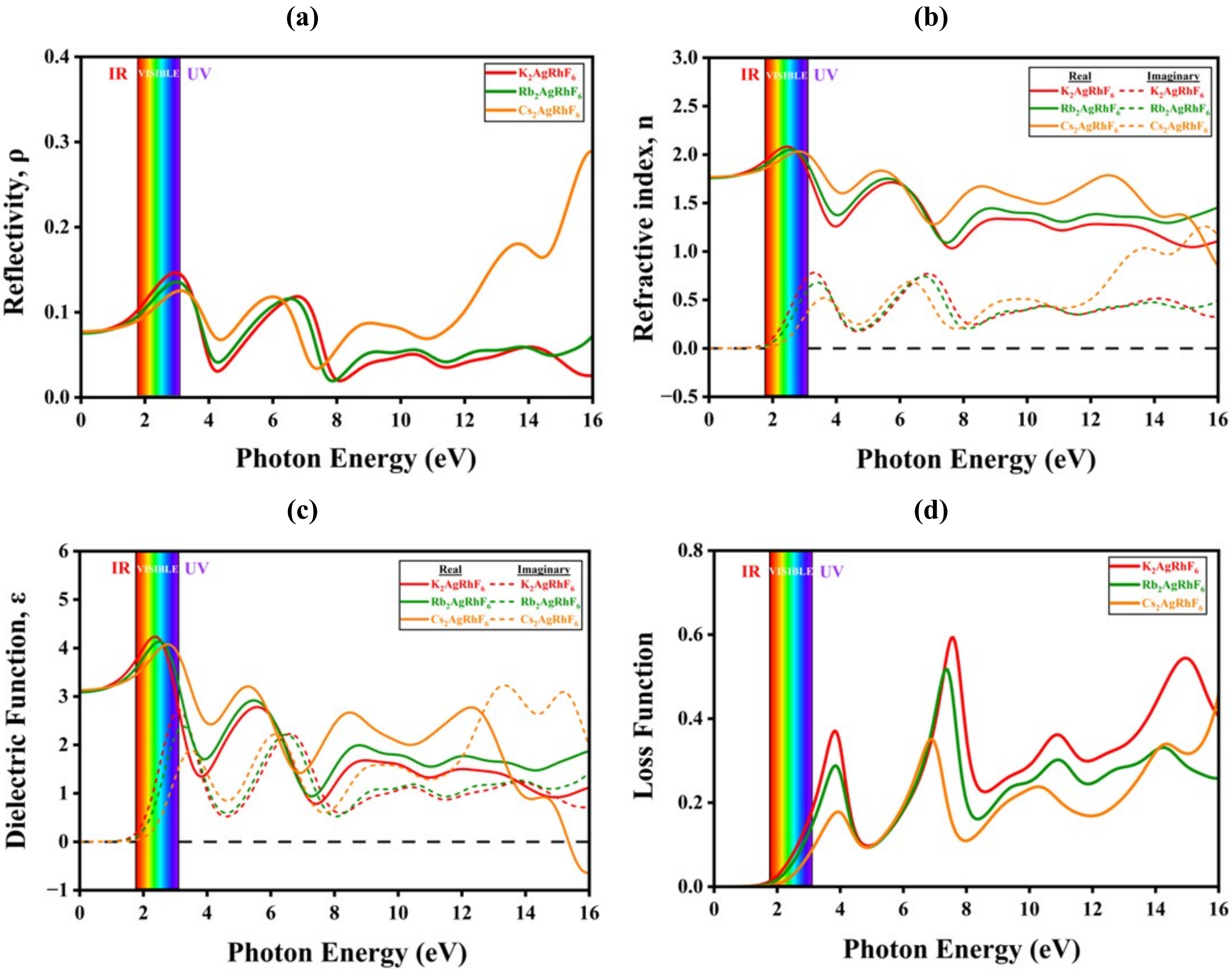

Fig. S4. Photon-energy-dependent optical properties of A_2AgRhF_6 double perovskites: (a) reflectivity, (b) refractive index, (c) dielectric function, and (d) loss function.

1.5. Loss Function, L(ω)

The electron energy-loss function (EELF), **L(ω)**, describes the energy dissipated by fast electrons traversing a material and is strongly associated with collective excitations such as plasmons and inter-band transitions, providing insight into the high-energy electronic response and dielectric screening behavior of solids [12]. It is expressed as,

$$L(\omega) = \frac{\varepsilon_2(\omega)}{\varepsilon_1^2(\omega) + \varepsilon_2^2(\omega)} \tag{S6}$$

The calculated spectra (Fig. S4(d)) show a weak onset at 2.5 to 3.0 eV, corresponding to the initiation of inter-band transitions and low-energy plasmon-like excitations in the near-UV region. A more pronounced peak occurs around 3.5 to 4.0 eV, where K_2AgRhF_6 and Rb_2AgRhF_6 exhibit higher intensities than Cs_2AgRhF_6, indicating a relatively stronger dielectric response. The dominant bulk plasmon resonance appears within 6.5 to 7.0 eV, with K_2AgRhF_6 showing the highest intensity, suggesting well-defined collective oscillations with relatively low damping. Additional broader features between 9 to 12 eV arise from higher-energy electronic transitions involving deeper valence states. The systematic variation from K → Rb → Cs reflects the influence of increasing A-site ionic size on band dispersion and dielectric screening, leading to slight shifts in plasmon energy and intensity. Low- and mid-energy plasmons (3 to 7 eV) indicate potential suitability for photonic devices, optical sensing, and surface-enhanced spectroscopies, while higher-energy excitations suggest possible relevance for deep-UV optoelectronic and plasmon-assisted energy conversion applications [13].

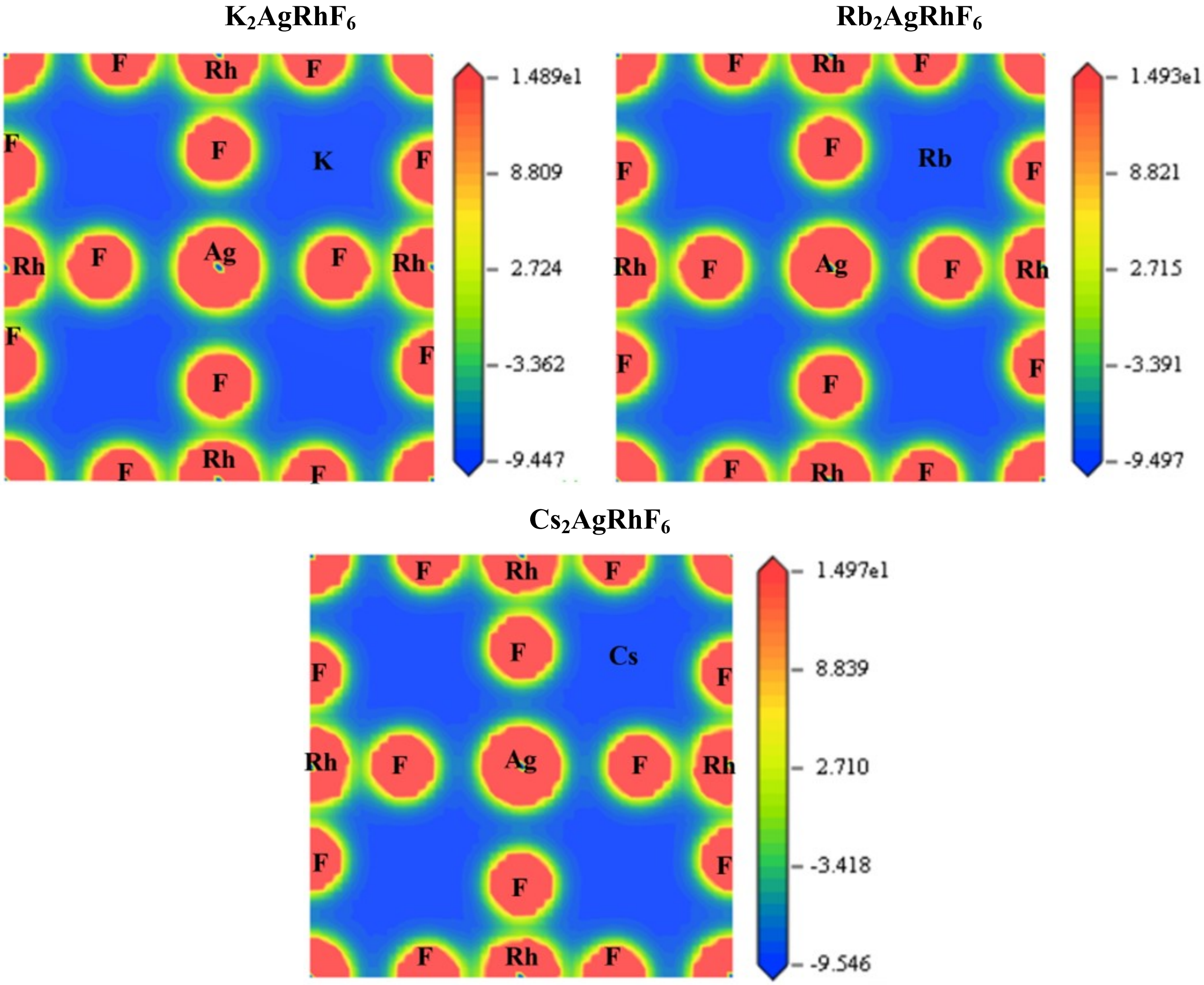

Fig. S5. Charge density mapping of A_2AgRhF_6 double perovskite materials.

$$(C_{11} - C_{12} > 0,\ C_{11}+2C_{12} > 0,\ C_{44} > 0) \qquad \text{(S7)}$$

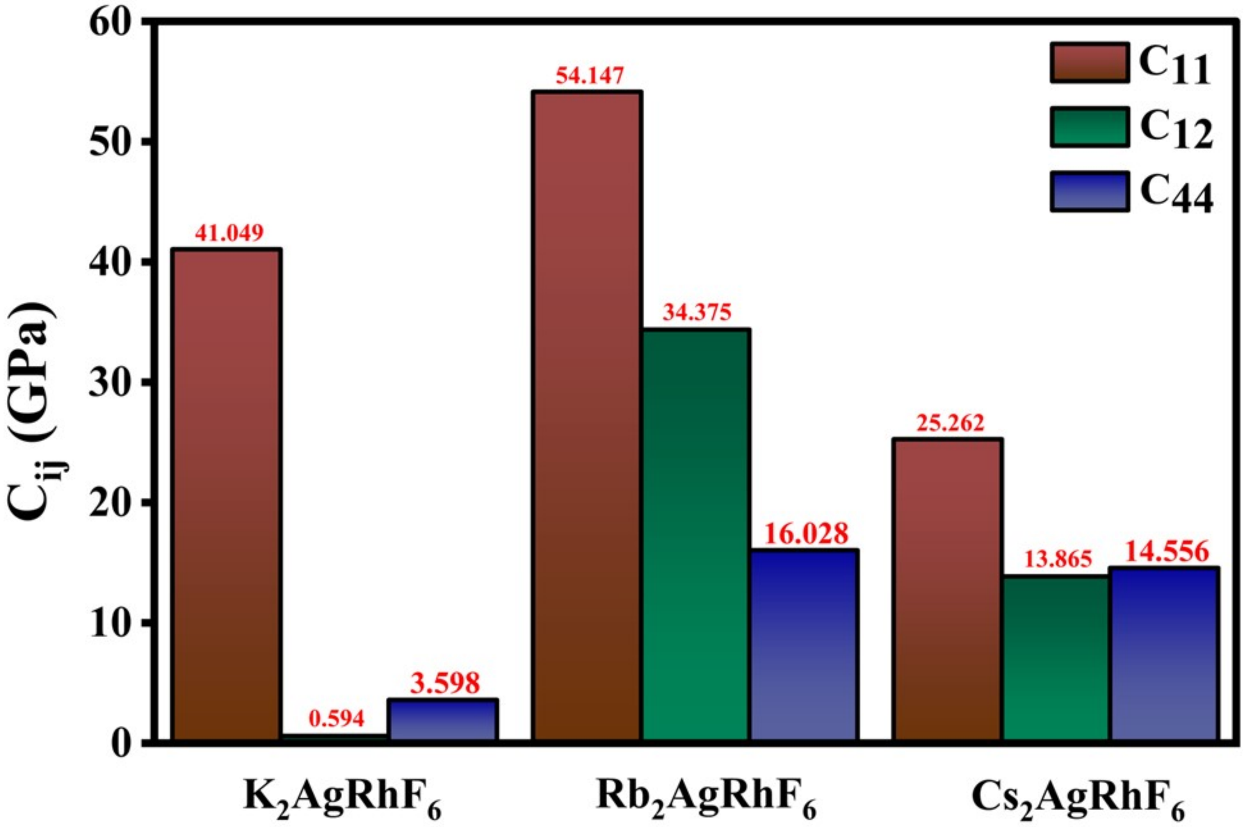

Fig. S6. Elastic constants and Born stability verification of cubic A_2AgRhF_6 double perovskites.

$$B = \frac{C_{11} + 2C_{12}}{3} \qquad \text{(S8)}$$

$$G_H = \frac{G_V + B_R}{2} \qquad \text{(S9)}$$

$$Y = \frac{9BG}{(3B + G)} \qquad \text{(S10)}$$

(a)

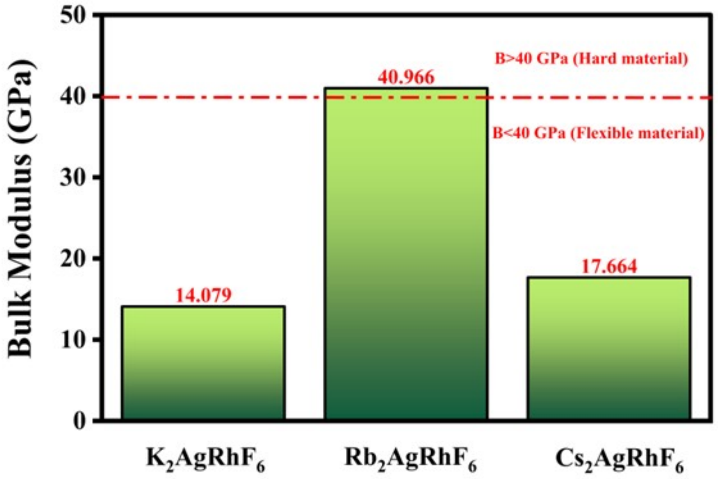
Bulk Modulus (GPa)
B>40 GPa (Hard material)
B<40 GPa (Flexible material)
40.966
14.079
17.664
K$_2$AgRhF$_6$
Rb$_2$AgRhF$_6$
Cs$_2$AgRhF$_6$

(b)

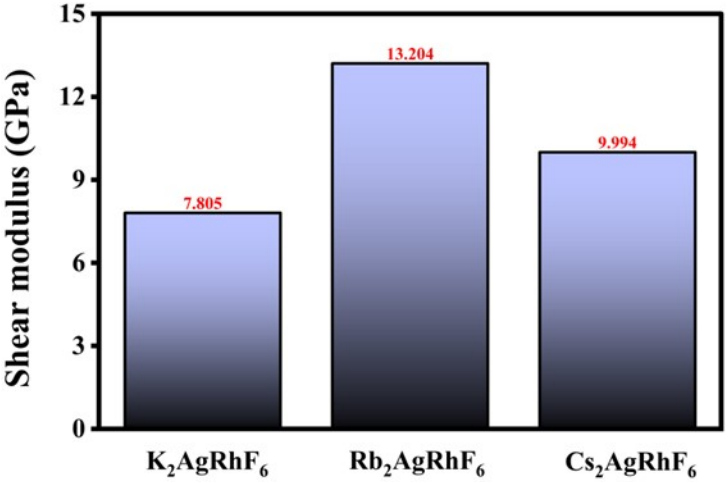
Shear modulus (GPa)
13.204
7.805
9.994
K$_2$AgRhF$_6$
Rb$_2$AgRhF$_6$
Cs$_2$AgRhF$_6$

(c)

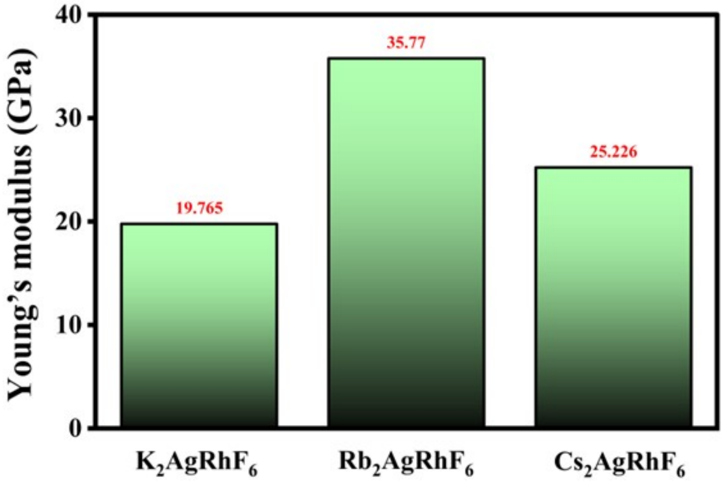
Young's modulus (GPa)
35.77
19.765
25.226
K$_2$AgRhF$_6$
Rb$_2$AgRhF$_6$
Cs$_2$AgRhF$_6$

(d)

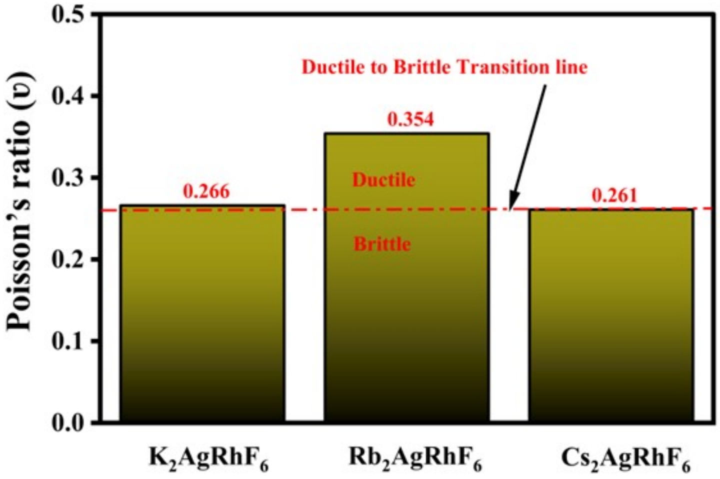
Poisson's ratio (υ)
Ductile to Brittle Transition line
0.354
0.266
0.261
Ductile
Brittle
K$_2$AgRhF$_6$
Rb$_2$AgRhF$_6$
Cs$_2$AgRhF$_6$

(e)

(f)

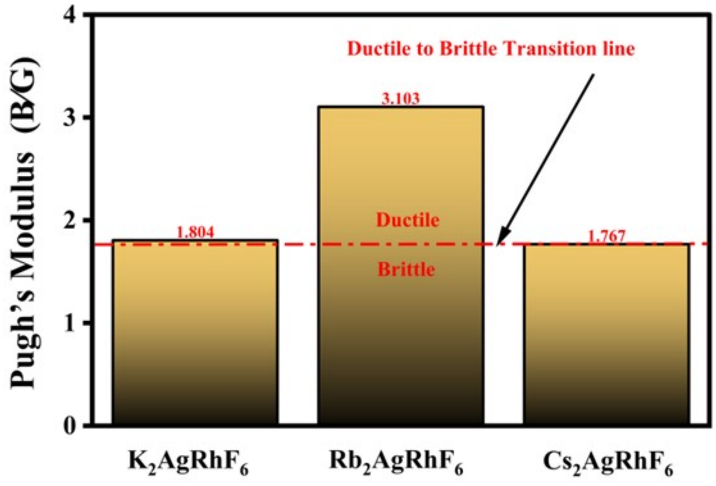

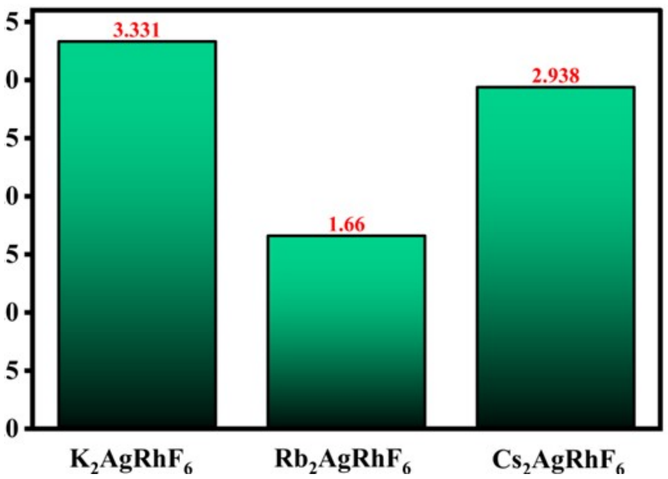

(g) **(h)**

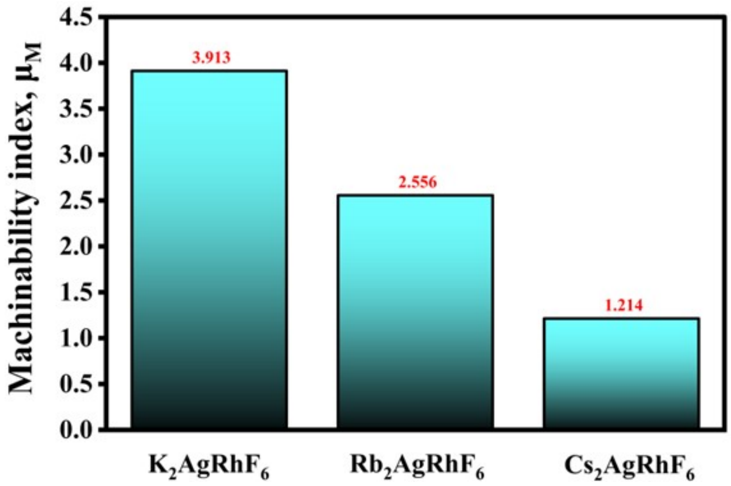

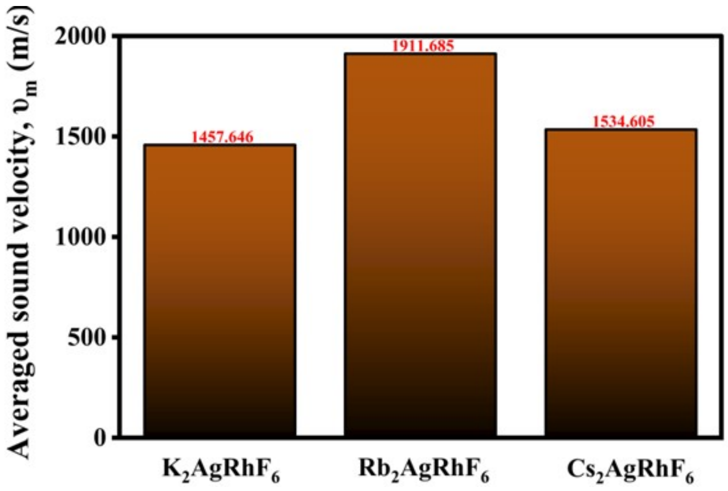

Fig. S7. Calculated mechanical and elastic properties of cubic double-perovskite fluorides A_2AgRhF_6 (a) bulk modulus, (b) shear modulus, (c) Young's modulus, (d) Poisson's ratio indicating ductile-brittle behavior, (e) Pugh's ratio (B/G), (f) Vickers hardness, (g) machinability index, and (h) average sound velocity.

$$v = \frac{3B - 2G}{2(3B + G)} \quad \text{(S11)}$$

$$B/G \quad \text{(S12)}$$

$$H_V = \frac{(1 - 2v)}{6(1 + v)} \quad \text{(S13)}$$

$$\mu_M = \frac{B}{C_{44}} \quad \text{(S14)}$$

$$A = \frac{2C_{44}}{C_{11} - C_{12}} \tag{S15}$$

$$A^U = \frac{B_V}{B_r} + 5\frac{G_V}{B_R} - 6 \geq 0 \tag{S16}$$

$$A^{eq} = \left(1 + \frac{5}{12}A^U\right) + \sqrt{(1 + \frac{5}{12}A^U)^2 - 1} \tag{S17}$$

$$A_G = \frac{G_V - G_R}{G_V + G_R} \tag{S18}$$

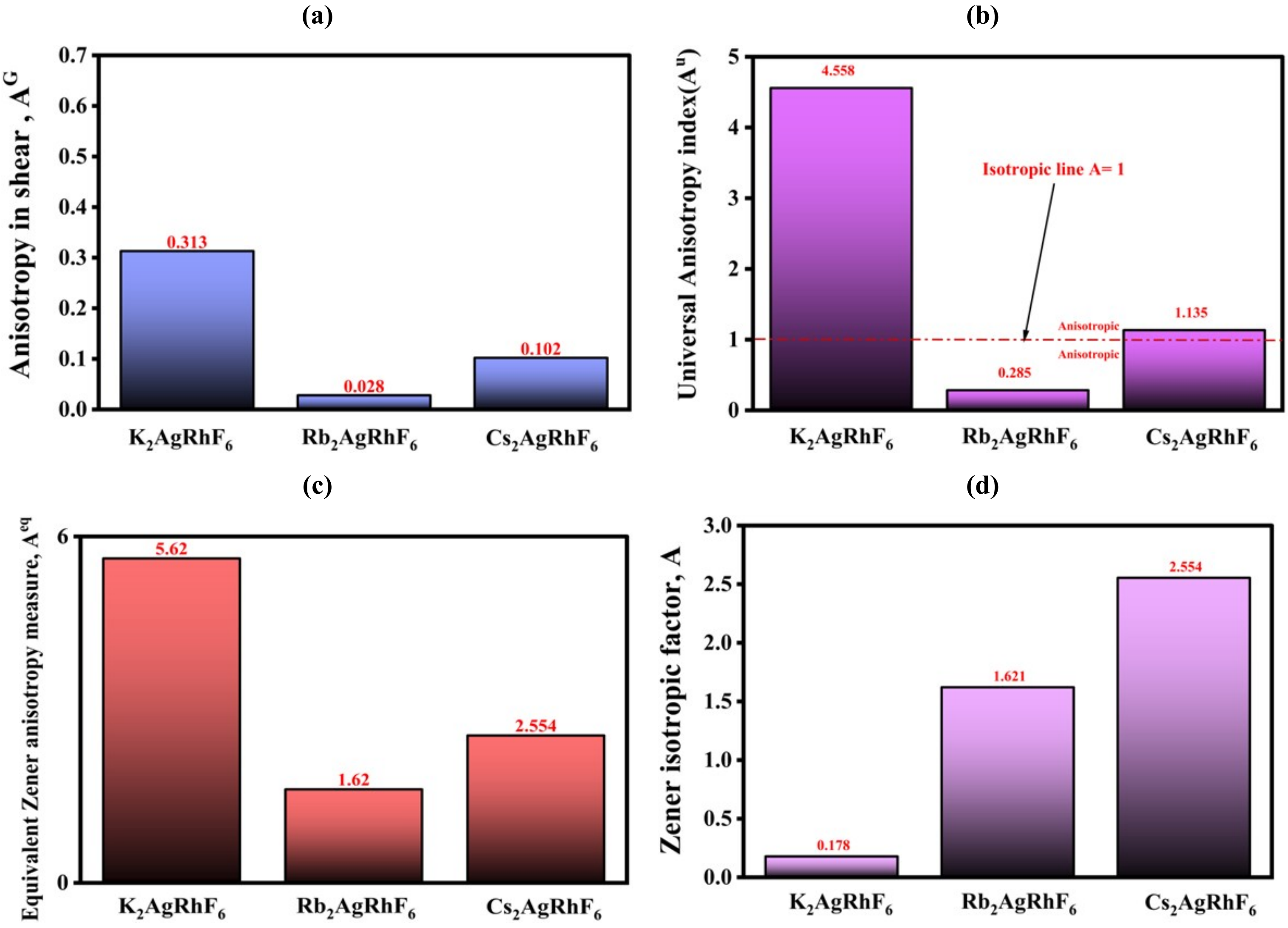

Fig. S8. Elastic anisotropy parameters (a) anisotropy in shear, (b) universal anisotropy index, (c) equivalent Zener anisotropy, and (d) zener isotropic factor for cubic A_2AgRhF_6 double perovskites.

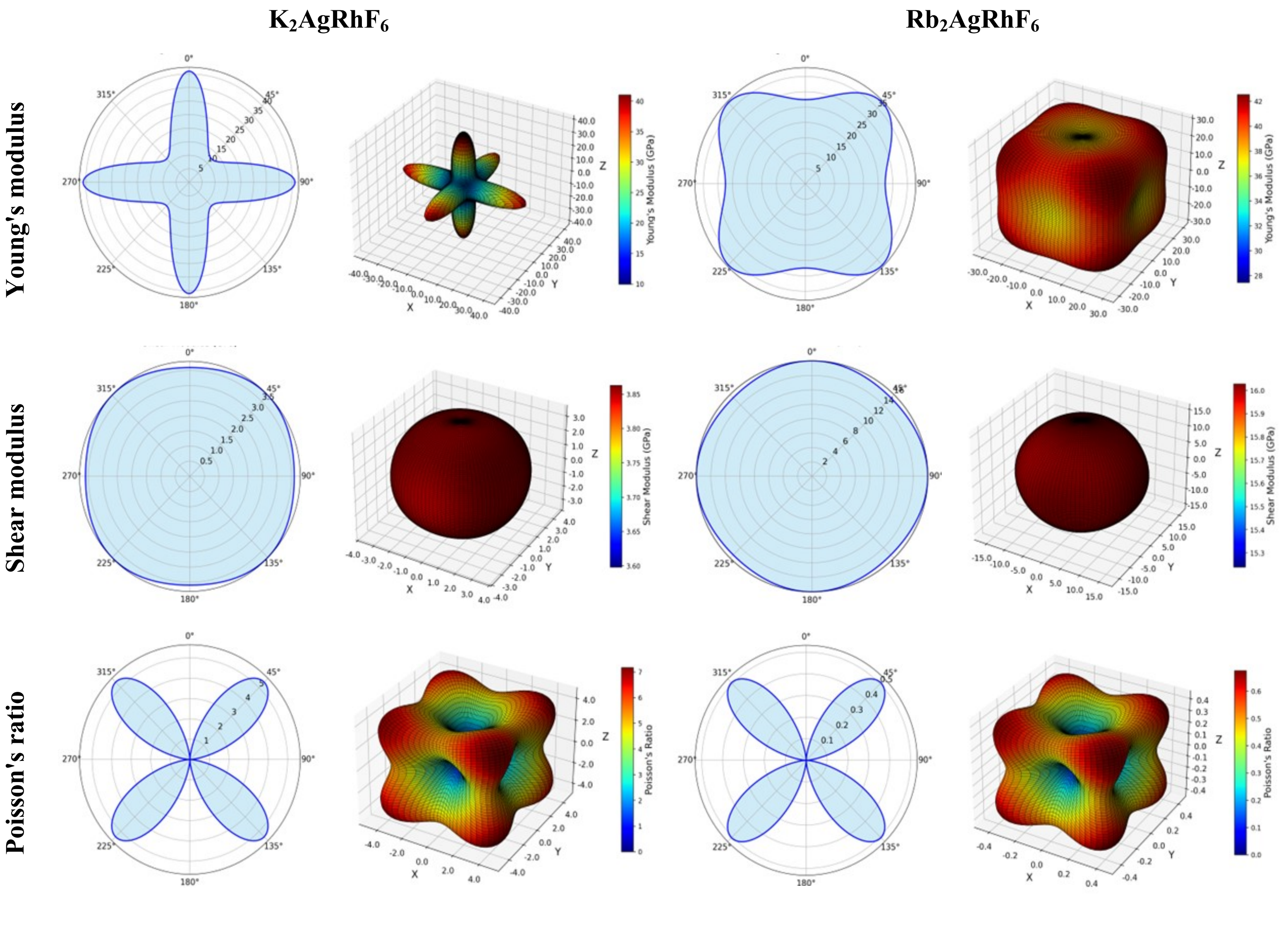

X-Y Plane 2D **3D** **X-Y Plane 2D** **3D**

Fig. S9. Graphical assessment of anisotropy showing 2D and 3D figures.

Cs_2AgRhF_6

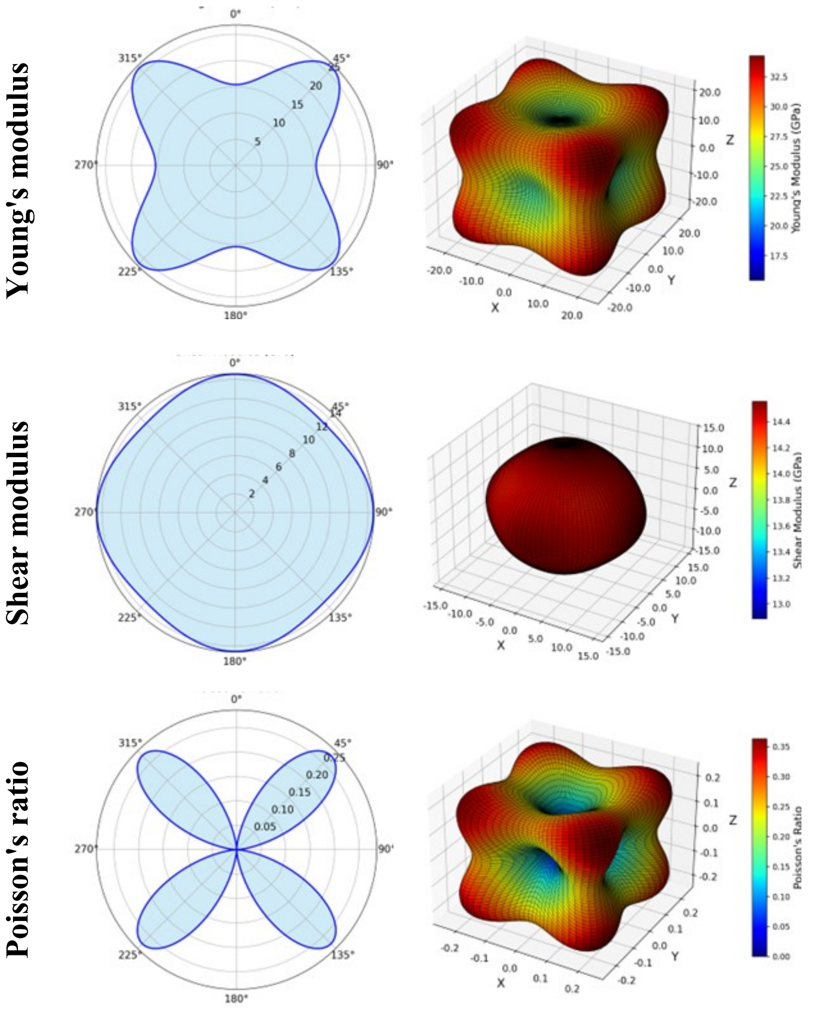

X-Y Plane 2D **3D**

Fig. S10. Graphical assessment of anisotropy showing 2D and 3D figures.

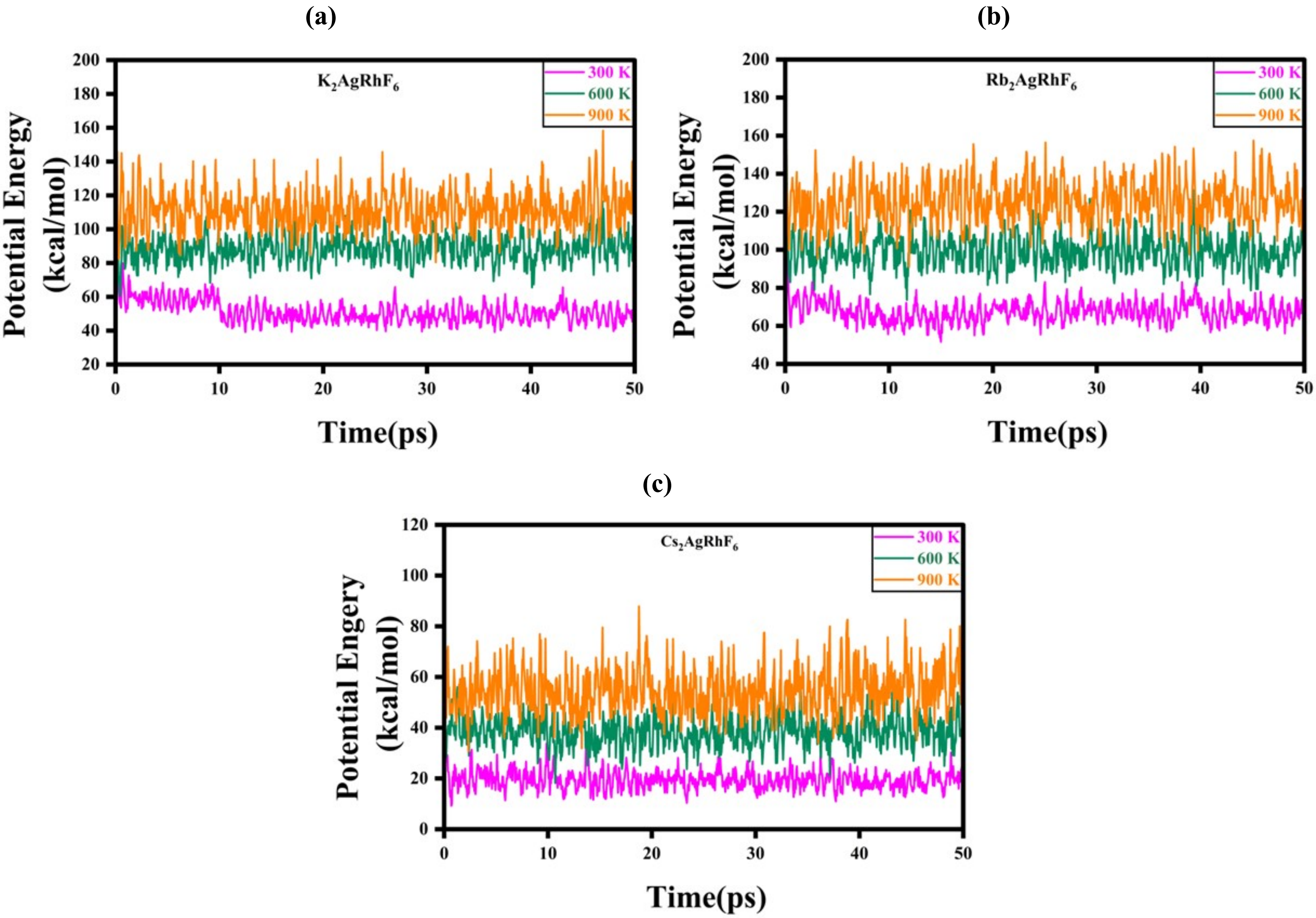

Fig. S11. Potential energy (kcal/mol) vs time for (a) K_2AgRhF_6, (b) Rb_2AgRhF_6, and (c) Cs_2AgRhF_6 at 300, 600, and 900 K, showing temperature-dependent fluctuations and stability.

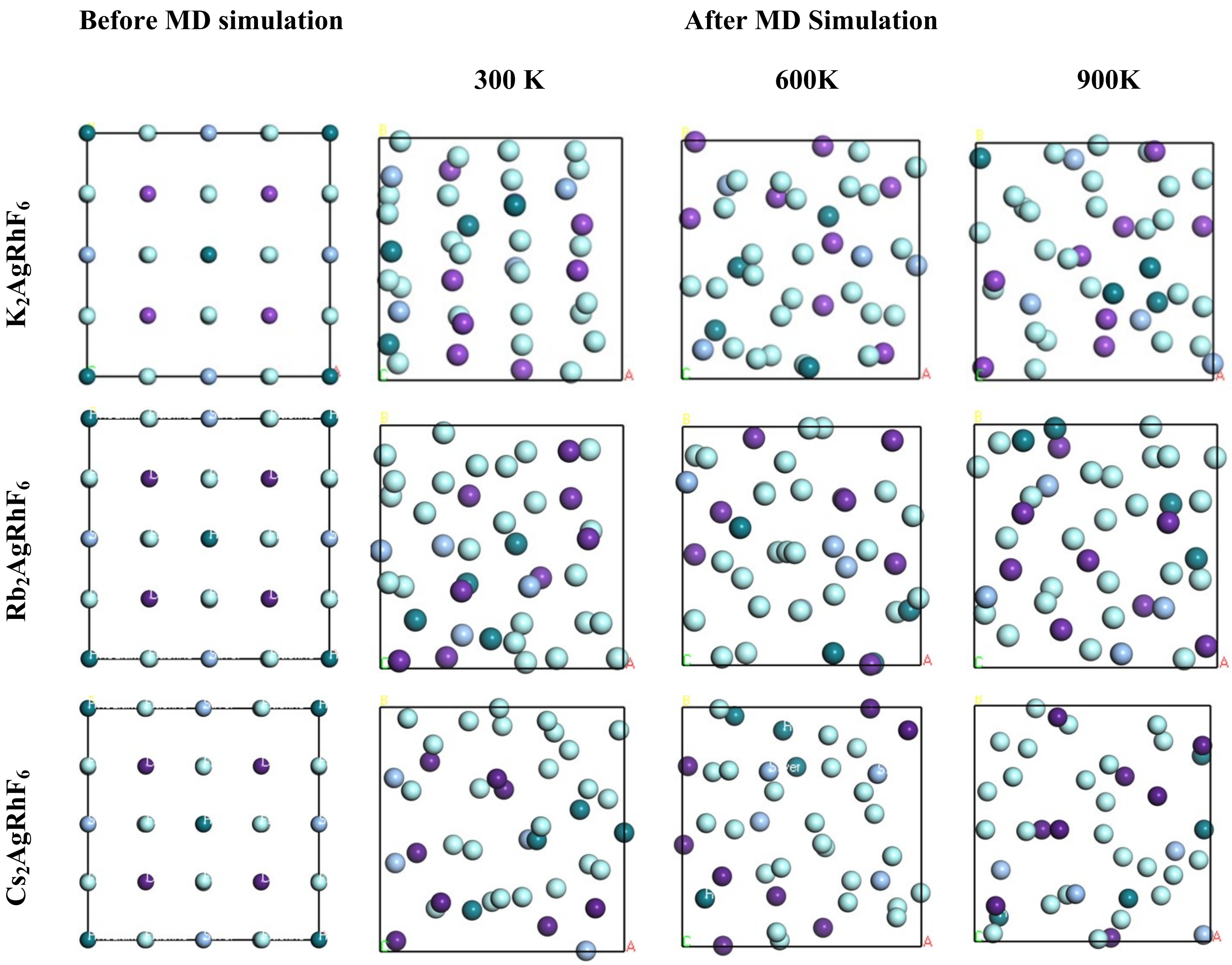

Fig. S12. Effect of Temperature on the Structural Stability of A_2AgRhF_6 (A = K, Rb, Cs) Double Perovskites

Table S2. Mulliken and Hirshfeld charge analysis of different atoms of A_2AgRhF_6 (Where A= Na, K, Cs) Perovskites.

Compound	Change spilling	Species	Mulliken atomic populations					Mulliken change	Hirshfeld change
			s	p	d	f	Total		
K_2AgRhF_6	0.09%	K	2.07	5.99	0.06	0.00	8.12	0.88	0.30
		Ag	2.23	6.39	9.93	0.00	18.55	0.45	0.38
		Rh	2.37	6.50	7.37	0.00	16.24	0.76	0.38
		F	1.95	5.55	0.00	0.00	7.50	-0.50	-0.24
Rb_2AgRhF_6	0.09%	Rb	2.09	5.94	0.10	0.00	8.13	0.87	0.26
		Ag	2.22	6.38	9.94	0.00	18.54	0.46	0.40
		Rh	2.37	6.51	7.37	0.00	16.24	0.76	0.39
		F	1.95	5.54	0.00	0.00	7.49	-0.49	-0.25
Cs_2AgRhF_6	0.10%	Cs	2.13	5.88	0.15	0.00	8.16	0.84	0.30
		Ag	2.20	6.35	9.96	0.00	18.51	0.49	0.43
		Rh	2.37	6.49	7.36	0.00	16.22	0.78	0.40
		F	1.95	5.54	0.00	0.00	7.49	-0.49	-0.25

Table S4. Input parameters used in the SCAPS-1D device simulations for the proposed solar cell (FTO/CdS/A_2AgRhF_6 (Where A= K, Rb, Cs) structure.

Parameters	FTO [14]	SnS_2 [15]	K_2AgRhF_6	Rb_2AgRhF_6	Cs_2AgRhF_6
Thickness (nm)	50	50	800	800	800
Band gap, E_g (eV)	3.6	2.24	1.194	1.158	1.077
Dielectric permittivity, ϵ_r	10	10.0	3.103	3.088	3.131
Electron affinity, χ (eV)	4.5	4.24	3.906	3.942	4.023
V_B effective density of states, N_V (cm^{-3})	2.48×10^{19}	2.2×10^{18}	1.51×10^{19}	1.478×10^{19}	1.532×10^{19}
C_B effective density of states, N_C (cm^{-3})	2×10^{18}	1.8×10^{19}	7.576×10^{18}	6.261×10^{18}	6.117×10^{18}
Donor density, N_D (cm^{-3})	1×10^{18}	1×10^{18}	0	0	0

Acceptor density, N_A (cm^{-3})	0	0	1 ×10^{17}	1 ×10^{17}	1 ×10^{17}
Electron mobility, μ_n (cm^2 V^{-1} s^{-1})	20	100	39.1	44.6	45.3
Hole mobility, μ_h (cm^2 V^{-1} s^{-1})	100	100	24.7	25.3	24.5
Defect density, N_t (cm^{-3})	1 ×10^{14}	1 × 10^{14}	1 × 10^{14}	1 × 10^{14}	1 × 10^{14}

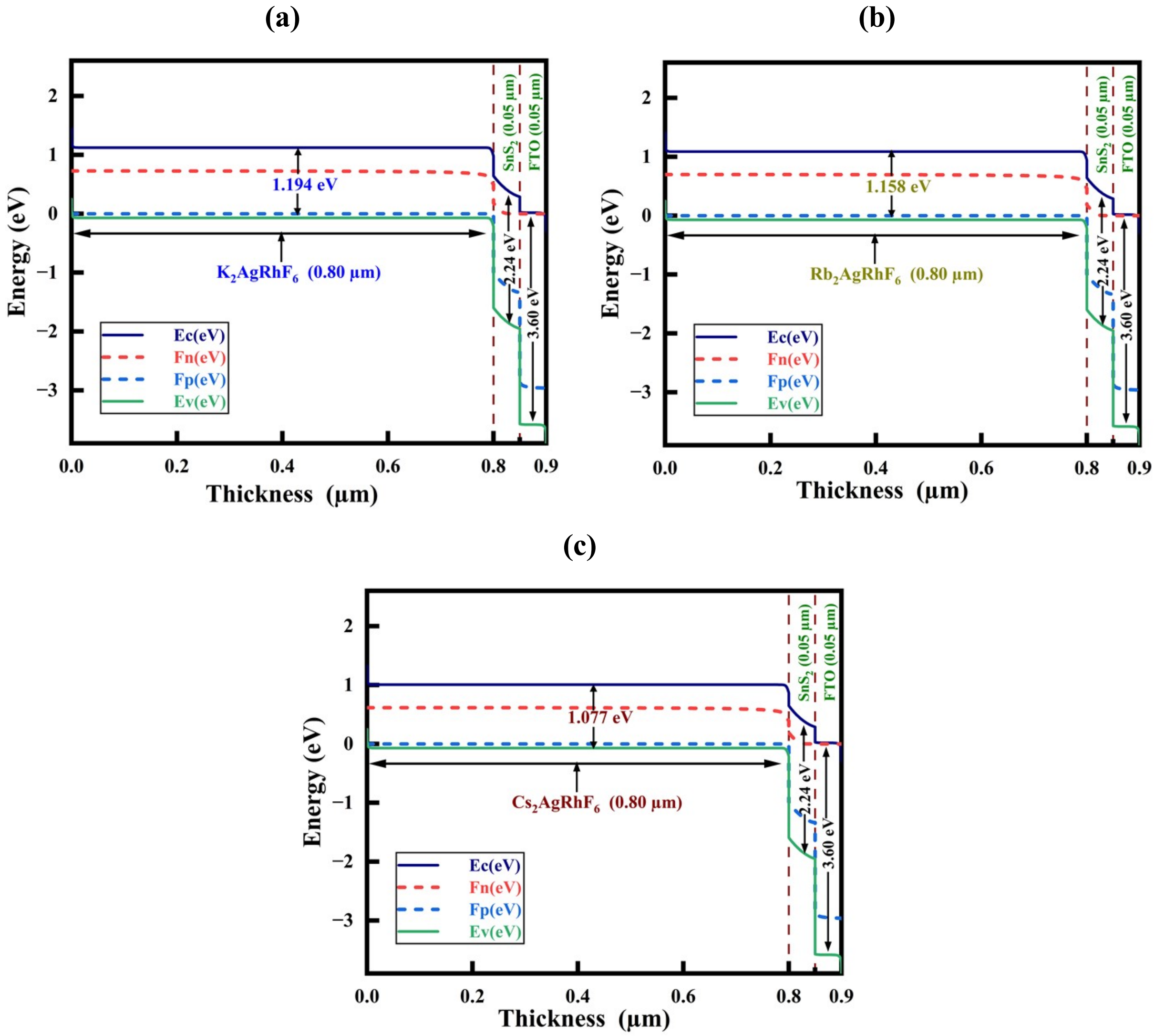

Fig. S13. Energy band diagrams of FTO/SnS_2/A_2AgRhF_6 (A = K, Rb, Cs) solar cell structures obtained from SCAPS-1D simulations: (a) K_2AgRhF_6, (b) Rb_2AgRhF_6, and (c) Cs_2AgRhF_6.

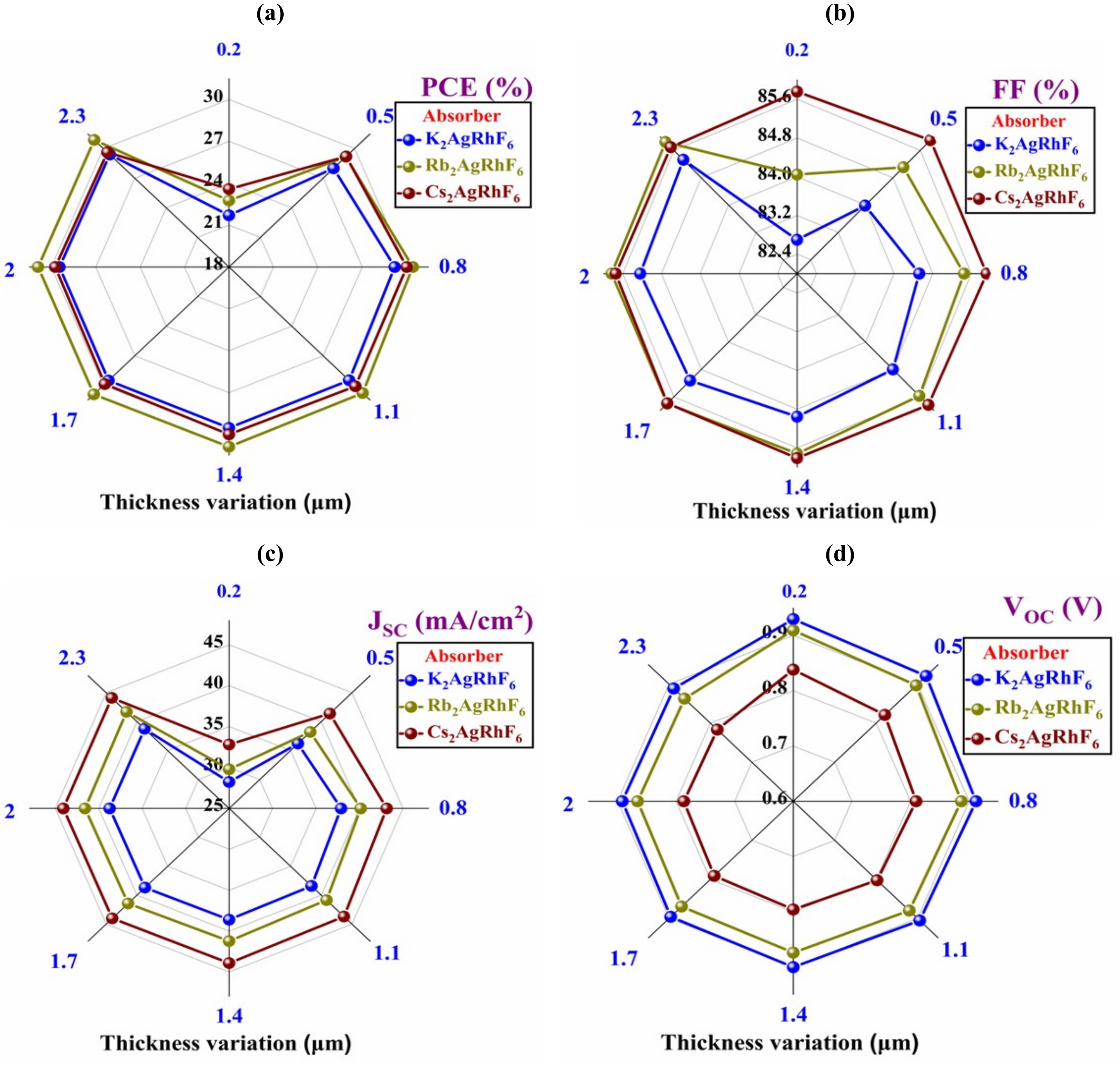
(a)
PCE (%)
Absorber
K2AgRhF6
Rb2AgRhF6
Cs2AgRhF6
0.2
0.5
0.8
1.1
1.4
1.7
2
2.3
30
27
24
21
18
Thickness variation (µm)
(b)
FF (%)
Absorber
K2AgRhF6
Rb2AgRhF6
Cs2AgRhF6
85.6
84.8
84.0
83.2
82.4
Thickness variation (µm)
(c)
JSC (mA/cm2)
Absorber
K2AgRhF6
Rb2AgRhF6
Cs2AgRhF6
45
40
35
30
25
Thickness variation (µm)
(d)
VOC (V)
Absorber
K2AgRhF6
Rb2AgRhF6
Cs2AgRhF6
0.9
0.8
0.7
0.6
Thickness variation (µm)

Fig. S14. Variation of (a) PCE, (b) FF, (c) J_{SC}, and (d) V_{OC} with absorber thickness (0.2 to 2.3 μm) for A_2AgRhF_6 (A = Na, K, Cs) based solar cells.

(a) **(b)**

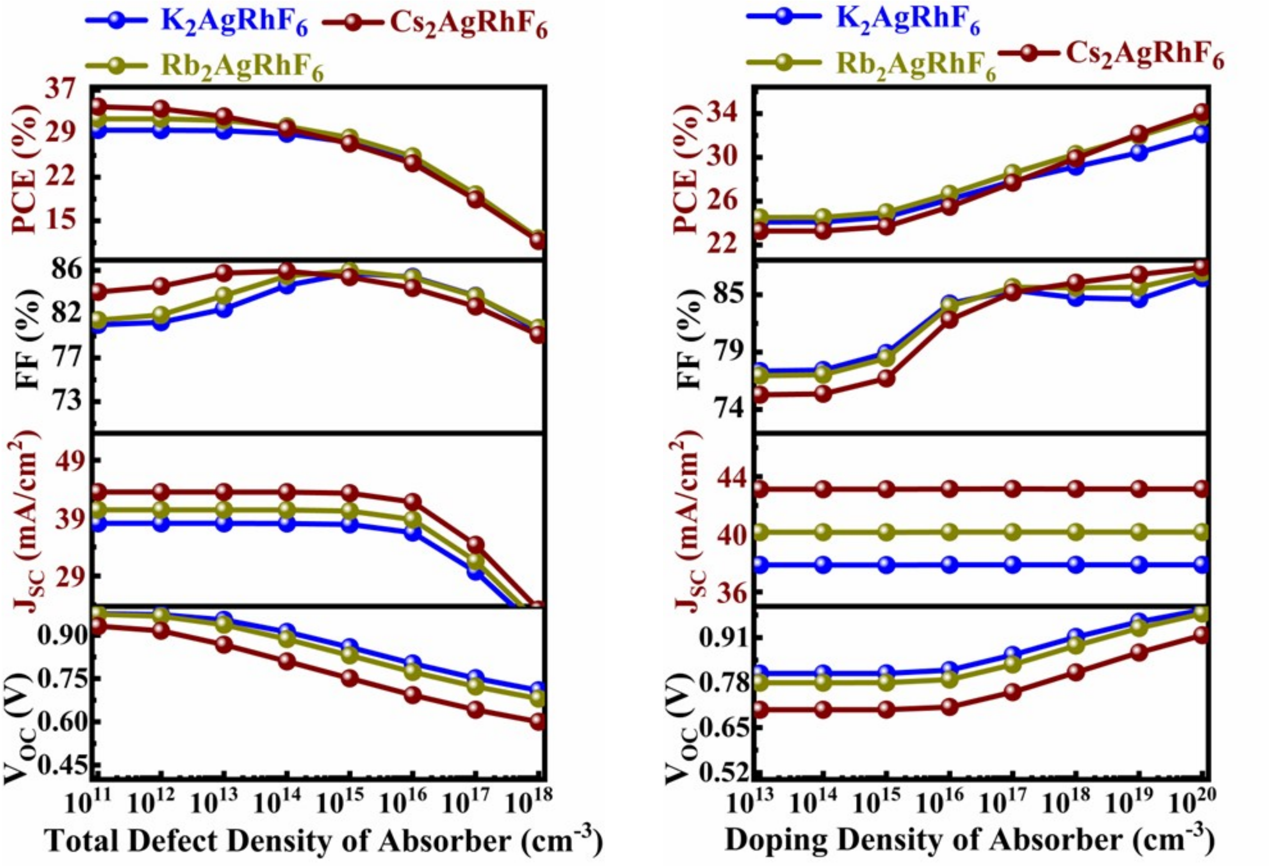

Fig. S15. Effect of absorber (a) defect density and (b) doping density on the photovoltaic parameters PCE, FF, J_{SC}, and V_{OC} of A_2AgRhF_6 (A = K, Rb, Cs) double perovskite solar cells.

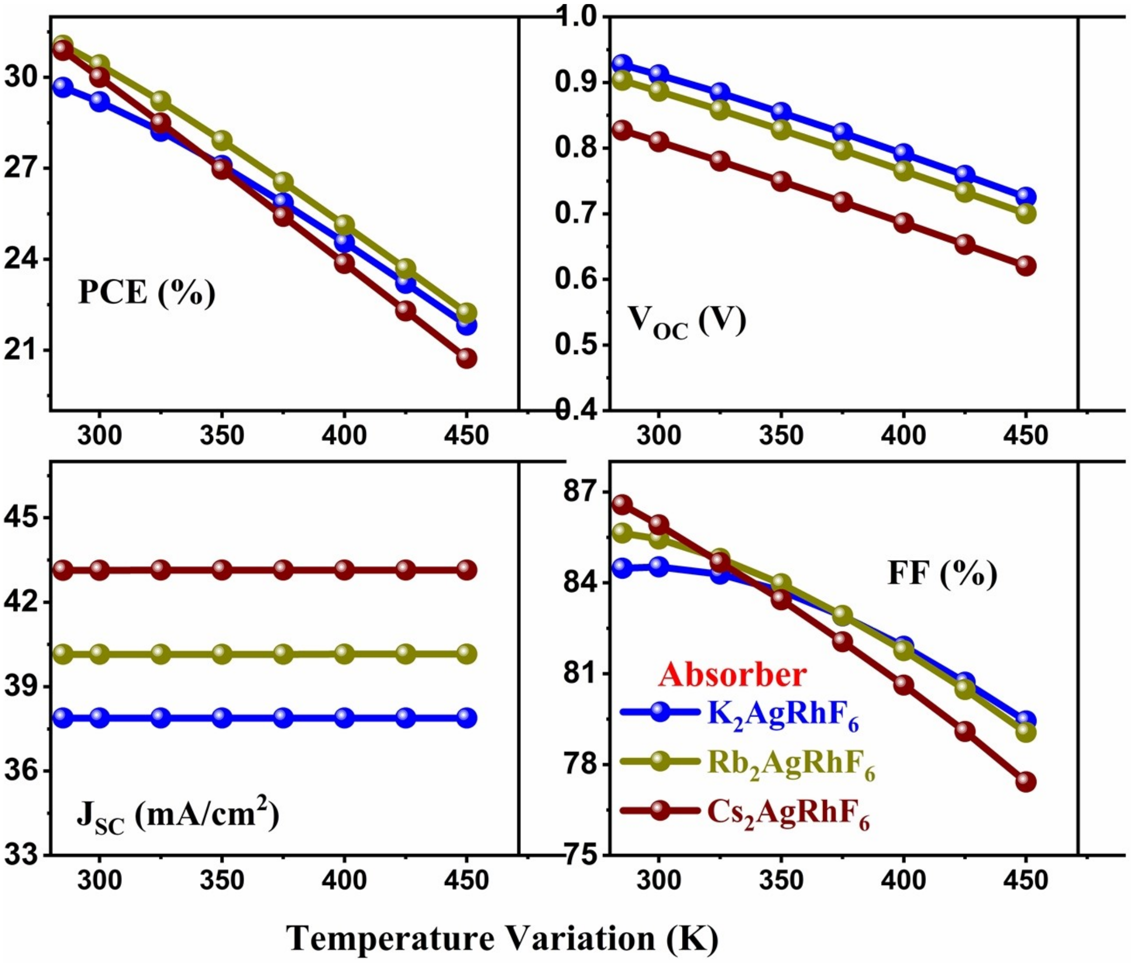

Fig. S16. Temperature-dependent photovoltaic parameters (PCE, V_{OC}, J_{SC}, and FF) of A_2AgRhF_6 (A = Na, K, Cs) double perovskite solar cells in the 285–450 K range.

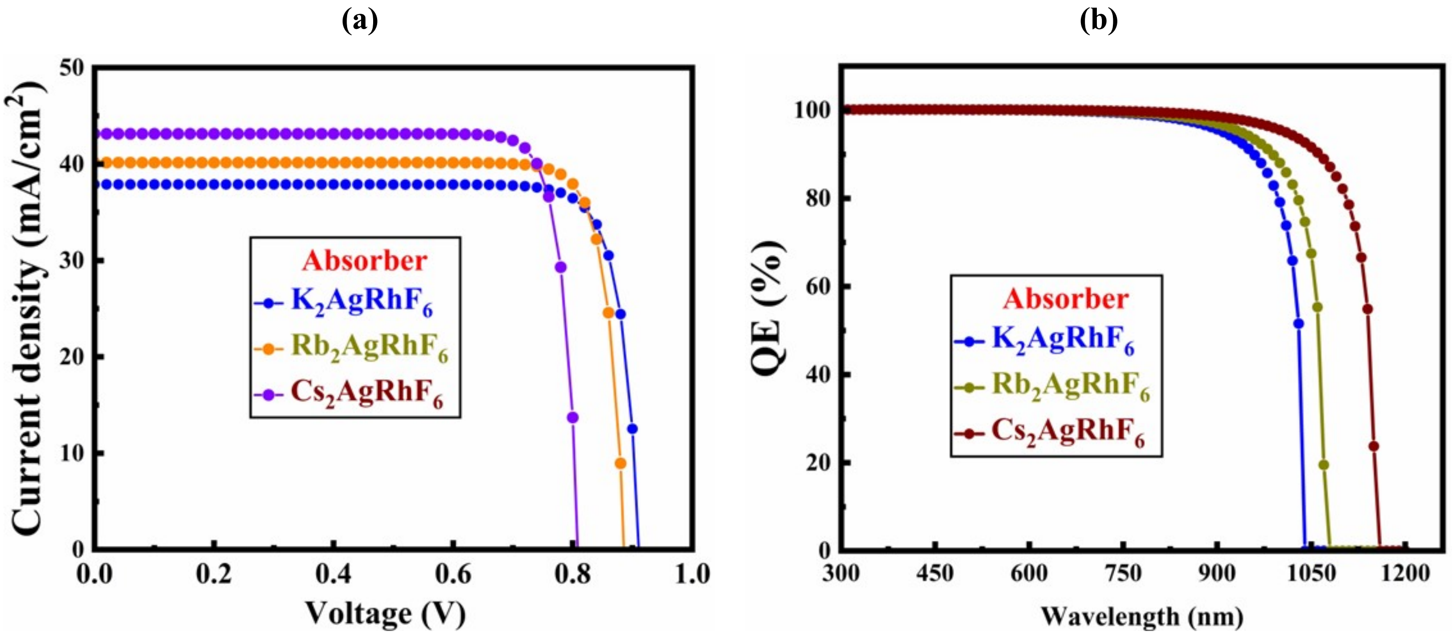

Fig. S17. (a) JV and (b) QE spectra of A_2AgRhF_6 double perovskite based solar cell

References

1 A. M. Jafar, K. A. Khalaph and H. B. Al Husseini, *Phys. Scr.*, 2022, 97, 085509.
2 M. Roknuzzaman, C. Zhang, K. Ostrikov, A. Du, H. Wang, L. Wang and T. Tesfamichael, *Sci Rep*, 2019, 9, 718.
3 S. A. Dar, G. Murtaza, T. Zelai, G. Nazir, H. Alkhaldi, H. Albalawi, N. A. Kattan, M. Irfan, Q. Mahmood and Z. Mahmoud, *Colloids and Surfaces A: Physicochemical and Engineering Aspects*, 2023, 664, 131145.
4 S. A. Aldaghfag, A. Aziz, A. Younas, M. Yaseen, A. Murtaza and H. H. Hegazy, *Journal of Solid State Chemistry*, 2022, 312, 123179.
5 I. A. Apon, Md. A. Hossain, R. Rafiu, Md. S. Hasan Saikot, Md. A. Rahman, J. R. Rajabathar, I. Boukhris, H. Albalawi, K. Kriaa and N. Elboughdiri, *Physica B: Condensed Matter*, 2025, 717, 417836.
6 V. Milman, K. Refson, S. J. Clark, C. J. Pickard, J. R. Yates, S.-P. Gao, P. J. Hasnip, M. I. J. Probert, A. Perlov and M. D. Segall, *Journal of Molecular Structure: THEOCHEM*, 2010, 954, 22–35.
7 T. Atsue, I. B. Ogunniranye and O. E. Oyewande, *Materials Science in Semiconductor Processing*, 2021, 133, 105963.
8 N. Delavari and M. Jafari, *Solid State Communications*, 2018, 275, 1–7.
9 R. Rafiu, K. Kriaa, Md. A. Rahman, I. A. Apon, S. AlFaify, C. Maatki, Md. S. Hasan and N. Elboughdiri, *Eur. Phys. J. Plus*, 2026, 141, 32.
10 P. G. R. Achary, S. K. Dehury and R. N. P. Choudhary, *J Mater Sci: Mater Electron*, 2018, 29, 6805–6816.
11 G. A. Samara, *Phys. Rev.*, 1966, 151, 378–386.

12 A. Dixit, J. Annie Abraham, M. Manzoor, M. Altaf, Y. Anil Kumar and R. Sharma, *Materials Science and Engineering: B*, 2024, 307, 117530.
13 M. R. Hasan, I. A. Apon and Md. Alamgir Hossain, *AIP Advances*, 2025, 15, 035032.
14 M. A. Rahman, A. Ghosh, N. S. Awwad, N. Elboughdiri, A. M. Alsuhaibani, Q. Mohsen and M. S. Refat, *Journal of Physics and Chemistry of Solids*, 2025, 203, 112728.
15 M. A. I. Bhuiyan, M. S. Reza, A. Ghosh, H. Al-Dmour, Y. A. Kumar, M. I. I. Rahim, M. Aktarujjaman, F. Yeasmin, H. Al-Lohedan, R. J. Ramalingam and M. S. Reza, *Optics Communications*, 2025, 583, 131761.
